# Flexibility of Metal-Organic Framework Tunable by Crystal Size at the Micrometer to Submillimeter Scale for Efficient Xylene Isomer Separation

**DOI:** 10.34133/2019/9463719

**Published:** 2019-10-17

**Authors:** Xiao Yang, Hao-Long Zhou, Chun-Ting He, Zong-Wen Mo, Jia-Wen Ye, Xiao-Ming Chen, Jie-Peng Zhang

**Affiliations:** MOE Key Laboratory of Bioinorganic and Synthetic Chemistry, School of Chemistry, Sun Yat-Sen University, Guangzhou 510275, China

## Abstract

Understanding, controlling, and utilizing the flexibility of adsorbents are of great importance and difficulty. Analogous with conventional solid materials, downsizing to the nanoscale is emerging as a possible strategy for controlling the flexibility of porous coordination polymers (or metal-organic frameworks). We report a unique flexibility controllable by crystal size at the micrometer to submillimeter scale. Template removal transforms [Cu_2_(pypz)_2_]·0.5*p*-xylene (MAF-36, Hpypz = 4-(1*H*-pyrazol-4-yl)pyridine) with one-dimensional channels to *α*-[Cu_2_(pypz)_2_] with discrete small cavities, and further heating gives a nonporous isomer *β*-[Cu_2_(pypz)_2_]. Both isomers can adsorb *p*-xylene to give [Cu_2_(pypz)_2_]·0.5*p*-xylene, meaning the coexistence of guest-driven flexibility and shape-memory behavior. The phase transition temperature from *α*-[Cu_2_(pypz)_2_] to *β*-[Cu_2_(pypz)_2_] decreased from ~270°C to ~150°C by increasing the crystal size from the micrometer to the submillimeter scale, ca. 2-3 orders larger than those of other size-dependent behaviors. Single-crystal X-ray diffraction showed coordination bond reconstitution and chirality inversion mechanisms for the phase transition, which provides a sufficiently high energy barrier to stabilize the metastable phase without the need of downsizing to the nanoscale. By virtue of the crystalline molecular imprinting and gate-opening effects, *α*-[Cu_2_(pypz)_2_] and *β*-[Cu_2_(pypz)_2_] show unprecedentedly high *p*-xylene selectivities of 16 and 51, respectively, as well as ultrafast adsorption kinetics (<2 minutes), for xylene isomers.

## 1. Introduction

As an indispensable raw material for polyethylene terephthalate and polybutylene terephthalate, *p*-xylene (pX) is generally obtained from xylene mixtures produced in catalytic reforming of crude oil. Because of their similar physical properties, the separation of xylene mixtures is challenging. The dominant industrial method for separating pX from xylene isomers is physical adsorption using FAU-type zeolites X and Y, but they suffer from low pX selectivity and adsorption rate, requiring energy-intensive operational conditions such as elevated temperature (~180°C) and pressure (~9 bar) [1]. Therefore, developing new adsorbents with high pX selectivity and adsorption rate at ambient conditions is of paramount importance.

**Figure 1:**
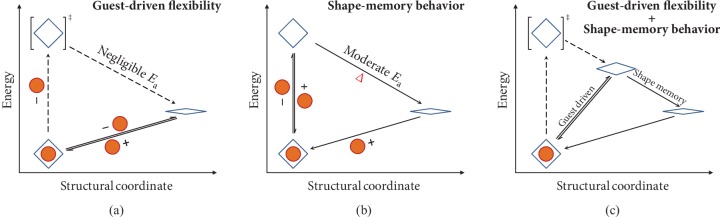
Thermodynamics/kinetics of guest-driven flexibility and shape-memory behavior. (a) Guest-driven flexibility. The guest-included open phase is thermodynamically stabilized by the host-guest interaction. There is a negligible energy barrier from the guest-free open phase to the closed phase, so that guest removal directly gives the closed phase. (b) Shape-memory behavior. There is a moderate energy barrier from the guest-free open phase to the closed phase, so that guest removal gives the metastable guest-free open phase, which can transform to the closed phase by heating. (c) Coexisting of guest-driven flexibility and shape-memory behavior (changes of the guest on the reaction direction are omitted for clarity).

Flexible adsorbents can change their structures in response to external stimuli, which have great potential to achieve ultrahigh separation efficiency [2–10], although successful examples for xylene isomers are still scarce [2–4]. Porous coordination polymers (PCPs), also known as metal-organic frameworks (MOFs), are the most remarkable type of flexible adsorbents [11–13]. Nevertheless, the framework flexibility of an adsorbent is difficult to design or control [5]. Structural transformations of flexible adsorbents are generally thermodynamically controlled (thermodynamically controlled flexibility (TCF)) and driven by guest change, where the thermodynamic potential of the host-guest system changes significantly (Δ*E*) by the host-guest interaction (Figure 1(a)). However, changing guest loading controls the host framework structure rather than framework flexibility or adsorption/separation behavior [5]. Previously, we demonstrated that the kinetic factor (kinetically controlled flexibility (KCF)) is critical for controlling framework flexibility [14]. With a suitable energy barrier (*E*_a_), structural transformation can be controlled by a physical stimulus/parameter such as temperature [14–19]. The energy barrier comprises the internal (intrinsic) part determined by host structure and the external (tunable) part dependent on particle structure such as crystal size, morphology, and defect.

The size-dependent properties emerging at the nanoscale are one of the most noteworthy developments of modern solid-state chemistry. As a subset of solid materials, MOFs may show similar behaviors, although known examples are still scarce [18–24]. Sakata et al. found that downsizing a flexible MOF to the nanoscale can increase the energy barrier to stabilize the open host configuration after guest removal, and heating (dependent on crystal size) can overcome the barrier to give the original closed configuration, mimicking the shape-memory behavior (Figure 1(b)) [18]. However, compared with conventional inorganic materials, it is much more difficult to downsize MOFs to the nanoscale. The relatively low stabilities of MOFs would be further emphasized at the nanoscale. The usefulness of size-dependent flexibility or shape-memory behavior for industrially important separation systems has not been demonstrated so far. Here, we report a MOF combining guest-driven flexibility and shape-memory behavior easily controllable by crystal size at the micrometer to submillimeter scale (Figure 1(c)), which is useful for pX separation.

## 2. Results

### 2.1. Synthesis, Structure, and Flexibility

**Figure 2:**
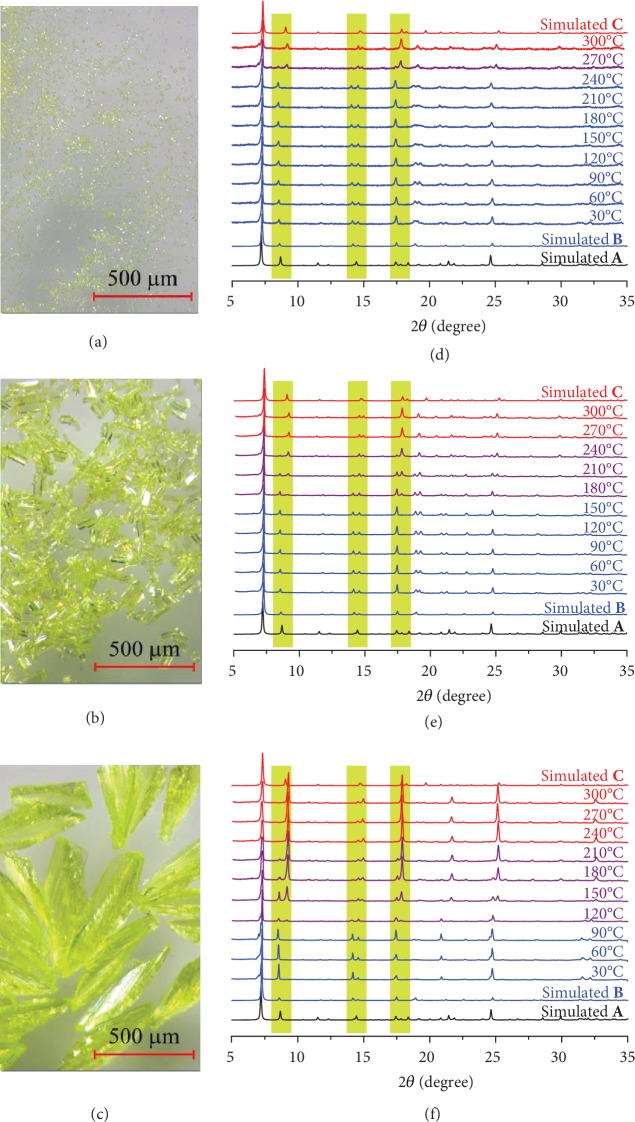
Crystal-size-dependent phase transition. (a–c) Optical images of **1**, **2**, and **3** and (d–f) variable-temperature PXRD patterns of **1**, **2**, and **3** after DCM extraction, respectively.

**Figure 3:**
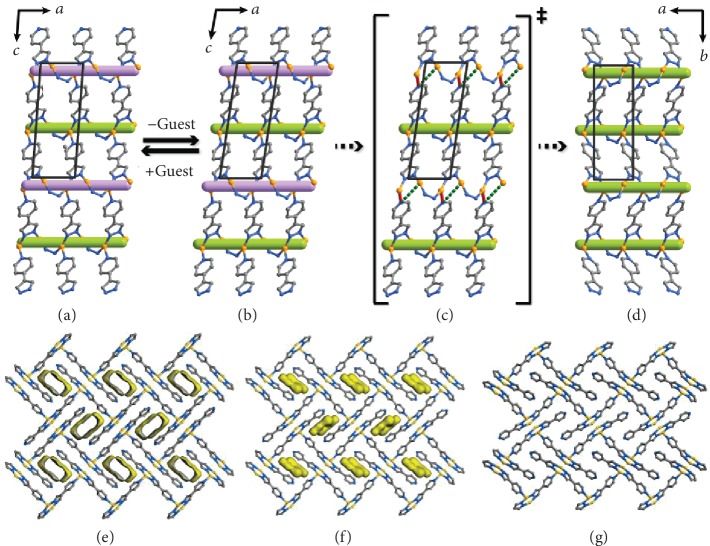
Single-crystal to single-crystal structural transformations. Coordination layers of (a) **A**, (b) **B**, (c) a proposed intermediate structure between **B** and **C**, and (d) **C**. Framework and pore surface structures of (e) **A**, (f) **B**, and (g) **C**.

Mixing an aqueous ammonia solution of [Cu(NH_3_)_2_]OH, a pyridine solution of 4-(1*H*-pyrazol-4-yl)pyridine (Hpypz), and pX at room temperature yielded yellow crystals of [Cu_2_(pypz)_2_]·0.5pX (MAF-36) [25]. Three samples with crystal sizes of around 0.5 × 0.5 × 1 *μ*m^3^, 30 × 30 × 100 *μ*m^3^, and 100 × 100 × 500 *μ*m^3^ (denoted as **1**, **2**, and **3**, respectively) were prepared by increasing the ammonia concentration (Figure 2(a)–(c) and [Supplementary-material supplementary-material-1]). Single-crystal X-ray diffraction revealed a zigzag-shaped two-dimensional (2D) coordination structure, in which antiparallel copper-pyrazolate 2_1_ helices are interconnected by Cu-pyridyl bonds in one of the two available directions (half metal ions and organic ligands become three-coordinated, others remain two-coordinated). These zigzag layers stack in the interdigitated mode to form the crystal, retaining zigzag 1D channels (void ratio = 18.5%, pore volume = 0.11 cm^3^ g^−1^) with a rectangle cross section filled with pX molecules (Figure 3(e), [Supplementary-material supplementary-material-1], and [Supplementary-material supplementary-material-1]).

Thermogravimetry (TG) curves of **1**, **2**, and **3** are very similar, which completely release pX molecules between 30 and 150°C, followed by a plateau until decomposition at about 310°C ([Supplementary-material supplementary-material-1]). The immersion of the three as-synthesized samples in dichloromethane (DCM) yielded a guest-free structure ([Supplementary-material supplementary-material-1]) with a powder X-ray diffraction (PXRD) pattern somewhat different from the as-synthesized structure. Interestingly, variable-temperature PXRD showed that the guest-free structure can transform to a new phase at high temperature, and the phase transition temperature is dependent on the crystal size (Figure 2(d)–(f) and [Supplementary-material supplementary-material-1]–[Supplementary-material supplementary-material-1]). In other words, we found three structures for [Cu_2_(pypz)_2_], i.e., the as-synthesized, guest-included structure [Cu_2_(pypz)_2_]·0.5pX (denoted as **A**) and the two guest-free frameworks *α*-[Cu_2_(pypz)_2_] and *β*-[Cu_2_(pypz)_2_] (denoted as **B** and **C**, respectively). The main differences among the PXRD patterns of **A**, **B**, and **C** appear at diffraction peaks at 8-9°, 14-15°, and 17-18°. For example, the diffraction peak at 8.6° shifts about -0.2° and +0.4° from **A** to **B** and from **A** to **C**, respectively. As shown in Figure 2(d)–(f), **1**, **2**, and **3** can retain phase **B** at up to 240°C, 150°C, and 90°C and completely transform to phase **C** at 300°C, 270°C, and 240°C, respectively, clearly demonstrating the crystal-size-dependent behavior at the micrometer to submillimeter scale. The stabilization of the metastable phase by crystal downsizing has been reported by three examples [18, 19], but they all require nanoscale crystals ([Supplementary-material supplementary-material-1]). Note that the transformations among **A**, **B**, and **C** combine the common guest-driven flexibility and the unique shape-memory behavior [16, 17]. In principle, the presence of an additional host configuration can provide more routes of structural transformations and more opportunities for controlling the guest responses. If the crystal size of MAF-36 is smaller than that of **1** or larger than that of **3**, the guest-free host would exhibit as just **B** or just **C** from room temperature to the decomposition temperature, giving pure guest-driven flexibility between **A** and **B** or between **A** and **C**, respectively. As shown in Figure 1, the shape-memory behavior of a flexible MOF can have more than one original structure (memory), in which the thermodynamic potential and the associated energy barrier sequentially decrease and increase, respectively.

### 2.2. Structural Transformation Mechanisms

Because the crystal sizes are large enough, we were able to clearly visualize the structural transformations by using single-crystal X-ray diffraction [26]. **A** and **B** possess the same *P*2_1_/*c* space group, but their unit-cell parameters have obvious differences ([Supplementary-material supplementary-material-1]), including 5.8% shortening of the *a*-axis, 4.4° increase of the *β*-angle, and 6.7% contraction of the unit-cell volume after guest removal. As reflected by the changes of the *a*-axis and the *β*-angle, the major difference between **A** and **B** lies in the deformation of the coordination layers (Figure 3(a) and (b)). The transformation from **A** to **B** is also accompanied by the slight swing of the uncoordinated pyridyl ends towards the inner pore, compressing and cutting the original 1D channel into discrete cavities (void ratio = 10.6%, pore volume = 0.06 cm^3^ g^−1^) with an irregular shape (Figure 3(f) and [Supplementary-material supplementary-material-1]).

The unit-cell volume of **C** further decreases by 6.3% from that of **B**. Interestingly, the orientations of the crystallographic *b*- and *c*-axes are altered. The local coordination geometry and network topology of **C** are basically the same as those of **A** and **B**. However, the chirality distribution of the copper-pyrazolate coordination helices in **C** is different from those of **A** and **B**. In the cases of **A** and **B**, right- and left-handed helices on the same layer are alternately arranged, giving a centrosymmetric coordination layer. In contrast, each layer in **C** is chiral because adjacent coordination helices on the same layer have the same chirality. Nevertheless, the 3D structure of **C** is still centrosymmetric because the layers are stacked in the heterochiral fashion. In other words, **A**/**B** and **C** possess syndiotactic (*P*,*M*,*P*,*M*) and heterotactic (*P*,*P*,*M*,*M*) chirality, respectively [27]. These structural differences suggest that in the structural transformation from **B** to **C**, half of the helices change their chirality. The opposite chirality of the helices in **B** and **C** mainly rely on the positions of the Cu ions and the pointing directions of the pyridyl ends.

Based on the minimum change principle, a possible mechanism for the transformation can be suggested: (1) the Cu-pyridyl bonds are broken to allow the Cu ions (originally three-coordinated, now two-coordinated) to move from one side to the other side of the layer and allow the pyridyl ends to swing to the opposite direction of the helix; meanwhile, (2) the neighbouring Cu ions (originally two-coordinated) move oppositely (versus the originally three-coordinated Cu ions) to reverse the chirality of the helix and (3) new Cu-pyridyl bonds form between the pyridyl ends and the originally two-coordinated Cu ions (Figure 3(c) and (d) and [Supplementary-material supplementary-material-1]). Such a chiral resolution also forces the deformation of the other parts of the 2D coordination layer. For example, the uncoordinated pyridyl end of the ligand further swings towards the inner channel to furnish a close packing structure (void ratio = 0%, Figure 3(g)). Obviously, PXRD analysis can hardly reveal these drastic local structural transformations including the cleavage/reformation of coordination bonds and chirality rearrangement.

Computational simulation showed that the framework energy of **B** is 16 kJ mol^−1^ higher than that of **C**, being consistent with their thermodynamic stabilities. The energy barrier of the transformation can be approximately estimated to be the energy needed to break the Cu-N coordination bond (calculated to be 103 kJ mol^−1^, [Supplementary-material supplementary-material-1]) [28]. This high internal/intrinsic energy barrier can explain the stability of **B** at the micrometer to submillimeter scale, without the need of the nanoscale.

### 2.3. Separation of Xylene Isomers

**Figure 4:**
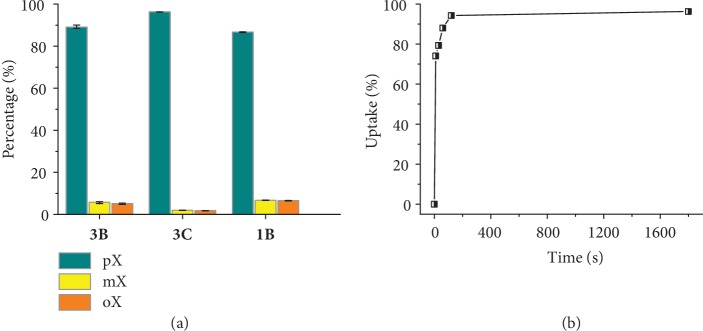
Separation performances for an equimolar mixture of xylene isomers. (a) Relative uptakes of xylene isomers in **3B**, **3C**, and **1B**. (b) Kinetic profile of the total uptake of xylene isomers in **1B**.

Since **A** can be regarded as a crystalline molecular-imprinted host templated by pX, high pX selectivity for xylene isomers can be expected [29, 30]. Crystals of as-synthesized **3** (**3A**) were treated to obtain pure **3B** and pure **3C** as the adsorbents with the same crystal size. TG and PXRD showed that **3B** and **3C** can achieve adsorption equilibrium within 3 and 24 h, respectively, after being immersed into each of the three xylene isomers (Figures [Supplementary-material supplementary-material-1]–[Supplementary-material supplementary-material-1]). At the same conditions, the uptakes of pX were close to saturation while those of *m*-xylene (mX) and *o*-xylene (oX) were only about half of that. To study the real separation performances, **3B** and **3C** were then immersed in an equimolar mixture of xylene isomers for 3 and 24 h, respectively. Gas chromatography analyses showed pX/mX/oX uptake ratios of 17(1) : 1.11(2) : 1 and 53.9(5) : 1.101(6) : 1 or pX selectivities of 16 and 51 for **3B** and **3C**, respectively (Figure 4(a) and [Supplementary-material supplementary-material-1]). These selectivities remained basically unchanged after three consecutive adsorption-desorption cycles (Figures [Supplementary-material supplementary-material-1]–[Supplementary-material supplementary-material-1]), demonstrating good reusability of **3B** and **3C**. For comparison, the highest pX adsorption selectivity reported so far was 7~10 for MOFs ([Supplementary-material supplementary-material-1]) [31–41] and 7.19 for the industrially used FAU-type zeolite [42].

Besides the molecular imprinting effect, the ultrahigh pX selectivities of **3B** and **3C** can be also attributed to their gate-opening type transformations during the adsorption processes [2], which give additional barriers for the adsorption of the less suitable guests mX and oX. Although **B** and **C** both transformed towards **A** after the adsorption of xylene isomers, **3B** was obviously easier/faster ([Supplementary-material supplementary-material-1]), indicating that a suitably higher gate-opening barrier can improve the guest separation selectivity.

Besides selectivity and recyclability, adsorption kinetics is also an important but often neglected factor. Considering that **3C** has ultrahigh pX selectivity but relatively slow adsorption rate, using **3B** with a slightly lower selectivity but much faster adsorption rate as the adsorbent may be more efficient for purifying pX (by using multiple adsorption-desorption cycles). Moreover, with a much smaller crystal size, the adsorption rate of **1B** is ultrafast (within 2 min, Figure 4(b) and [Supplementary-material supplementary-material-1]) and its selectivity is similar to that of **3B** (Figure 4(a) and [Supplementary-material supplementary-material-1]). To the best of our knowledge, MOF adsorbents reported in the literature require at least hours to achieve satisfactory batch adsorption of xylene isomers [2, 32, 33, 37]. It is worth noting that the crystal size of **1B** is still at the normal micrometer scale, which is advantageous for avoiding common problems of nanomaterials, such as instability and difficulty of synthesis.

## 3. Discussion

In conclusion, MAF-36 exhibits a unique flexibility among a guest-included and two guest-free states, in which the common guest-driven structural transformation and the special shape-memory behavior coexist, and noteworthily, the latter is tunable by crystal size at the micrometer to submillimeter scale. The structural transformation of MAF-36 from the less stable guest-free state to the more stable guest-free state requires the reconstitution of coordination bonds, which generates a high internal energy barrier (without the need of an external barrier) sufficient to stabilize the metastable phase at the normal crystal size (without the need of the nanoscale) and high temperature. Being proportional to the outer surface area of the crystal, surface energy is significant only at the nanoscale. The concentration of a crystal defect generally increases along with the crystal size, which explains the difficulty for growing large single crystals. Structural transformation is believed to occur first at the lattice defect [18, 43, 44], so that increasing crystal size can decrease the external energy barrier due to the increase of lattice defect in a single crystallite. Because the crystal sizes of MAF-36 are well above nanoscale, the contribution of surface energy should be negligible, so that of the lattice defect should play a dominant role in controlling the phase transition temperature. By contrast, the structural transformation from the guest-included state to the less stable guest-free state involves only distortion, which can have a negligible energy barrier to allow the reversible guest-driven actions.

MAF-36 also exhibits tunable and superb pX separation performances from xylene isomers, which can be explained by the molecular imprinting effect (pX as template and target guest) and the gate-opening effect (from the guest-free states to the guest-included state). The molecular imprinting effect has been well demonstrated in the field of amorphous organic polymers but has not been used in crystalline adsorbents (including MOFs) before. Adding target molecules in the synthesis/crystallization environment may yield host-guest type crystals templated by the target which exhibit high selectivity for the target [45]. However, to achieve the molecular imprinting effect similar to MAF-36, the host-guest type crystal needs to be robust for the template removal and reversible adsorption-desorption processes. Note that both rigid and flexible structures can be robust. The results of this work not only advance our understanding of the flexibility of MOFs but also open up a new avenue for future development of smart materials.

## 4. Materials and Methods

### 4.1. Materials and Measurements

All commercially available reagents and solvents were used as received without further purification. 4-(1*H*-Pyrazol-4-yl)pyridine (Hpypz) was prepared according to a method found in the literature [46]. Optical microscope images were recorded using a light transmission microscope (Olympus BX51, Melville, NY). Scanning electron microscopy (SEM) images were recorded on a JEOL JSM-6700 Field Emission SEM device. Thermogravimetry (TG) analyses were performed on a NETZSCH TG 209 F3 instrument with a ramp rate of 10.0°C min^−1^ under nitrogen atmosphere at ambient pressure. PXRD data were collected on a SmartLab X-ray powder diffractometer using a D/teX Ultra250 detector in the 5-35° (2*θ*) range in Cu K*α* reflection (K*β* characteristic X-rays being filtered by a K*β* filter (Ni foil)) and with a step size of 0.01° 2*θ* at room temperature. Variable-temperature PXRD measurements (with Anton Paar XRK 900 sample holder connected with TCU 750 temperature control unit) were performed under N_2_ flow (50 mL min^−1^, controlled by a mass flowmeter). The temperature ramp rate was 10°C min^−1^ during the change of measurement temperature. Elemental analyses (EA) were performed by a vario EL Elemental Analyzer (C, H, and N).

### 4.2. Syntheses

Under N_2_ atmosphere, a solution of Hpypz (0.435 g, 3 mmol) and *p*-xylene (5 mL) in pyridine (30 mL) was poured into a solution of [Cu(NH_3_)_2_]OH (0.332 mg, 1.5 mmol) in aqueous ammonia (30 mL) at room temperature. The mixture was left to stand for 1.5 hours, and then the precipitate was filtrated, washed with methanol, and dried in air to obtain microcrystalline samples. The ammonia concentration was 6.25%, 12.5%, and 25% for samples **1** (0.440 g, yield 62%), **2** (0.496 g, yield 70%), and **3** (0.514 g, yield 72%), respectively. EA calcd (%) for [Cu_2_(pypz)_2_]·0.5C_8_H_10_: C 51.28, H 3.66, N 17.94; found: C 51.06, H 3.41, N 18.17 for **1**, C 50.98, H 3.43, N 18.24 for **2**, C 51.05, H 3.44, N 18.24 for **3**. EA calcd (%) for *α*-[Cu_2_(pypz)_2_]: C 46.26, H 2.91, N 20.23; found: C 46.30, H 2.67, N 20.25 for **1**, C 46.37, H 2.63, N 20.38 for **2**, C 46.56, H 2.63, N 20.34 for **3**.

### 4.3. X-Ray Crystallography

Diffraction intensities were collected on a Pilatus XtaLAB P300D diffractometer with Mo K*α* radiation. Absorption corrections were applied using the multiscan program REQAB. The structures were solved with the direct method and refined with a full-matrix least-squares technique based on *F*^2^ with the SHELXTL program package. Anisotropic thermal parameters were applied to all nonhydrogen atoms. Hydrogen atoms were generated by the riding mode. Crystallographic data in CIF format have been deposited in the Cambridge Crystallographic Data Centre (CCDC) under deposition numbers 1548426–1548428. Data collection and structural refinement parameters are given in [Supplementary-material supplementary-material-1].

### 4.4. Computational Details

Periodic density functional theory (PDFT) calculations were performed through the Materials Studio 5.5 package. Optimization of the framework structures was carried out by the DMol^3^ module. The widely used generalized gradient approximation (GGA) with the Perdew-Burke-Ernzerhof (PBE) function and the double numerical plus d-function (DND) basis set as well as the DFT Semicore Pseudopots (DSPP) were used. The convergence tolerances were set as follows: energy, 2 × 10^−5^ kcal/mol; force, 1.0 × 10^−3^ kcal/mol/Å; and displacement, 1.0 × 10^−5^ Å.

### 4.5. Liquid-Phase Adsorption and Separation Experiments

In single-component adsorption experiments, the MOF sample (15 mg) was immersed in 100 *μ*L *o*-xylene, *m*-xylene, or *p*-xylene at room temperature. After 3 (**3B**) or 24 (**3C**) hours, the sample was filtrated and dried in the N_2_ flow (20 mL/min) at 30°C for 1 hour. The xylene uptakes were calculated by the weight loss below 180°C of the TG curves.

In the characterization of adsorption kinetics for ternary components, the MOF sample (25 mg) was immersed in 200 *μ*L of an equimolar mixture of xylene isomers at room temperature for a given time. The immersion times were 0.5, 1, 3, 6, 12, 24, and 48 hours for **3B** and **3C** and 10, 30, 60, 120, and 1800 seconds for **1B**. After that, the sample was filtrated and dried in the N_2_ flow (20 mL/min) at 30°C for 1 hour. The xylene uptakes were calculated by the weight loss below 180°C of the TG curves.

For determining the xylene adsorption ratios, the MOF sample (250 mg) was immersed in a 2 mL equimolar mixture of xylene isomers at room temperature for 0.5 (**1B**), 3 (**3B**), and 24 (**3C**) hours, respectively, and then filtered and dried in the N_2_ flow (20 mL/min) at 30°C for 1 hour. After that, 10 mg of sample was digested using 0.5 mL of an 8 M aqueous solution of HNO_3_. Xylene molecules in the digestion solution were extracted by *n*-heptane (1.5 mL, chromatographically pure) for two times. The digestion solution was gathered together for gas chromatography-mass spectrometry (GC-MS) analyses to determine the molar ratios of the isomers. To regenerate the adsorbents, xylene-loaded **1B** and **3B** were immersed in DCM for 1 day and xylene-loaded **3C** was further heated at 150°C after the same DCM extraction step. All adsorbents were evacuated at 70°C for 3 hours before immersing into the xylene mixture.

### 4.6. Gas Chromatography-Mass Spectrometry Analyses

GC-MS measurements were performed by an Agilent 7890A-5975C apparatus with a CP7502 capillary column (25 m × 0.35 mm i.d.). In each measurement, 1 *μ*L of analyte was injected to determine the molar ratio of the isomers. The conditions of the temperature settings are as follows: the column temperature is 70°C (held for 1 min), which increases to 150°C at 5°C/min (held for 1 min) and then cooled to 70°C; the front injection temperature is 210°C. The He flow was 3 mL/min, and the split mode was 10 : 1 for measured samples. The selectivity is *α*_ij_ = (*A*_i_/*A*_j_)/(*A*_io_/*A*_jo_), where *A*_i_ and *A*_j_ are integrated peak areas of xylene isomers adsorbed into the framework, calculated by the equipped analysis software, and *A*_io_ and *A*_jo_ are the integrated peak areas of xylene isomers of an equimolar ternary xylene mixture. Each reported selectivity value in this work is the average result of three parallel experiments and the standard deviation of which was also calculated. The *p*-xylene selectivity is defined according to the literature [31] as 2*q*_3_/(*q*_1_ + *q*_2_), where *q*_1_, *q*_2_, and *q*_3_ are uptakes of *o*-, *m*-, and *p*-xylene, respectively, in ternary adsorption experiments.

## Data Availability

All data needed to evaluate the conclusions in the paper are present in the paper and in the Supplementary Materials. Additional data related to this paper may be requested from the authors.

## Supplementary Materials

Supplementary Materials Table S1: crystal-size-dependent framework flexibility reported for MOFs. Table S2: crystallographic data and structure refinements. Table S3: comparison of pX adsorption selectivities reported for MOFs. Figure S1: SEM images of **A**. Figure S2: crystal structure of **A**. Figure S3: detailed framework pore structures of **A** and **B**. Figure S4: TG curves of **A**. Figure S5: PXRD patterns of [Cu_2_(pypz)_2_]·0.5pX after DCM extraction. Figure S6: magnified Figure 2(d). Figure S7: magnified Figure 2(e). Figure S8: magnified Figure 2(f). Figure S9: the full illustration of the proposed transformation mechanism from **B** to **C**. Figure S10: optimized structures and energies of the structural fragments involved in the transformation from **B** to **C**. Figure S11: TG curves of **3B** and **3C** after adsorption of xylene isomers. Figure S12: PXRD patterns of **3B** and **3C** after having been loaded with xylene isomers. Figure S13: adsorption kinetic profiles of **3B** and **3C** for an equimolar mixture of xylene isomers. Figure S14: gas chromatography curve of the *n*-heptane solution containing an equimolar mixture of xylene isomers. Figure S15: xylene selectivities of **3B** in three consecutive ad-/desorption cycles. Figure S16: xylene selectivities of **3C** in three consecutive ad-/desorption cycles. Figure S17: TG curves for **1B** after having been immersed in an equimolar mixture of xylene isomers for different times. Figure S18: xylene selectivities of **1B** in three consecutive ad-/desorption cycles.

## References

[1] Yang Y., Bai P., Guo X. (2017). Separation of xylene isomers: a review of recent advances in materials. *Industrial & Engineering Chemistry Research*.

[2] Mukherjee S., Joarder B., Manna B., Desai A. V., Chaudhari A. K., Ghosh S. K. (2015). Framework-flexibility driven selective sorption of *p*-xylene over other isomers by a dynamic metal-organic framework. *Scientific Reports*.

[3] Zhang W. Y., Lin Y. J., Han Y. F., Jin G. X. (2016). Facile separation of regioisomeric compounds by a heteronuclear organometallic capsule. *Journal of the American Chemical Society*.

[4] Jie K., Liu M., Zhou Y. (2018). Near-ideal xylene selectivity in adaptive molecular pillar[n]arene crystals. *Journal of the American Chemical Society*.

[5] Zhang J. P., Zhou H. L., Zhou D. D., Liao P. Q., Chen X. M. (2018). Controlling flexibility of metal-organic frameworks. *National Science Review*.

[6] Jin H., Li Y. (2018). Flexibility of metal-organic frameworks for separations: utilization, suppression and regulation. *Current Opinion in Chemical Engineering*.

[7] Bereciartua P. J., Cantín Á., Corma A. (2017). Control of zeolite framework flexibility and pore topology for separation of ethane and ethylene. *Science*.

[8] Zhou Y. J., Jie K. C., Zhao R., Huang F. H. (2019). Cis-trans selectivity of haloalkene isomers in nonporous adaptive pillararene crystals. *Journal of the American Chemical Society*.

[9] Taylor M. K., Runčevski T., Oktawiec J. (2018). Near-perfect CO_2_/CH_4_ selectivity achieved through reversible guest templating in the flexible metal-organic framework Co(bdp). *Journal of the American Chemical Society*.

[10] Yang L., Cui X., Zhang Y., Yang Q., Xing H. (2018). A highly sensitive flexible metal-organic framework sets a new benchmark for separating propyne from propylene. *Journal of Materials Chemistry A*.

[11] Schneemann A., Bon V., Schwedler I., Senkovska I., Kaskel S., Fischer R. A. (2014). Flexible metal-organic frameworks. *Chemical Society Reviews*.

[12] Chang Z., Yang D. H., Xu J., Hu T. L., Bu X. H. (2015). Flexible metal-organic frameworks: recent advances and potential applications. *Advanced Materials*.

[13] Elsaidi S. K., Mohamed M. H., Banerjee D., Thallapally P. K. (2018). Flexibility in metal-organic frameworks: a fundamental understanding. *Coordination Chemistry Reviews*.

[14] Zhang J. P., Chen X. M. (2008). Exceptional framework flexibility and sorption behavior of a multifunctional porous cuprous triazolate framework. *Journal of the American Chemical Society*.

[15] Shivanna M., Yang Q. Y., Bajpai A. (2018). Readily accessible shape-memory effect in a porous interpenetrated coordination network. *Science Advances*.

[16] Kanoo P., Haldar R., Reddy S. K. (2016). Crystal dynamics in multi-stimuli-responsive entangled metal-organic frameworks. *Chemistry - A European Journal*.

[17] Zhu A. X., Yang Q. Y., Kumar A. (2018). Coordination network that reversibly switches between two nonporous polymorphs and a high surface area porous phase. *Journal of the American Chemical Society*.

[18] Sakata Y., Furukawa S., Kondo M. (2013). Shape-memory nanopores induced in coordination frameworks by crystal downsizing. *Science*.

[19] Kavoosi N., Bon V., Senkovska I. (2017). Tailoring adsorption induced phase transitions in the pillared-layer type metal-organic framework DUT-8(Ni). *Dalton Transactions*.

[20] Tanaka D., Henke A., Albrecht K. (2010). Rapid preparation of flexible porous coordination polymer nanocrystals with accelerated guest adsorption kinetics. *Nature Chemistry*.

[21] Hijikata Y., Horike S., Tanaka D. (2011). Differences of crystal structure and dynamics between a soft porous nanocrystal and a bulk crystal. *Chemical Communications*.

[22] Zhang C., Gee J. A., Sholl D. S., Lively R. P. (2014). Crystal-size-dependent structural transitions in nanoporous crystals: adsorption-induced transitions in ZIF-8. *Journal of Physical Chemistry C*.

[23] Tanaka S., Fujita K., Miyake Y. (2015). Adsorption and diffusion phenomena in crystal size engineered ZIF-8 MOF. *Journal of Physical Chemistry C*.

[24] Krause S., Bon V., Senkovska I. (2018). The effect of crystallite size on pressure amplification in switchable porous solids. *Nature Communications*.

[25] Li F., Lin R. B., Wei Y. S. (2014). Metal-ion controlled solid-state reactivity and photoluminescence in two isomorphous coordination polymers. *Inorganic Chemistry Frontiers*.

[26] Zhang J. P., Liao P. Q., Zhou H. L., Lin R. B., Chen X. M. (2014). Single-crystal X-ray diffraction studies on structural transformations of porous coordination polymers. *Chemical Society Reviews*.

[27] Wheaton Richard C. A., Puddephatt J. (2007). A coordination polymer of gold(I) with heterotactic architecture and a comparison of the structures of isotactic, syndiotactic, and heterotactic isomers. *Angewandte Chemie International Edition*.

[28] Luo Y. R. (2007). *Comprehensive Handbook of Chemical Bond Energies*.

[29] Chen L., Wang X., Lu W., Wu X., Li J. (2016). Molecular imprinting: perspectives and applications. *Chemical Society Reviews*.

[30] Yuan Y., Yang Y., Ma X. (2018). Molecularly imprinted porous aromatic frameworks and their composite components for selective extraction of uranium ions. *Advanced Materials*.

[31] Torres-Knoop A., Krishna R., Dubbeldam D. (2014). Separating xylene isomers by commensurate stacking of *p*-xylene within channels of MAF-X8. *Angewandte Chemie International Edition*.

[32] Warren J. E., Perkins C. G., Jelfs K. E. (2014). Shape selectivity by guest-driven restructuring of a porous material. *Angewandte Chemie, International Edition*.

[33] Huang W., Jiang J., Wu D., Xu J., Xue B., Kirillov A. M. (2015). A highly stable nanotubular MOF rotator for selective adsorption of benzene and separation of xylene isomers. *Inorganic Chemistry*.

[34] Gee J. A., Zhang K., Bhattacharyya S. (2016). Computational identification and experimental evaluation of metal-organic frameworks for xylene enrichment. *The Journal of Physical Chemistry C*.

[35] Lannoeye J., van de Voorde B., Bozbiyik B., Reinsch H., Denayer J., de Vos D. (2016). An aliphatic copper metal-organic framework as versatile shape selective adsorbent in liquid phase separations. *Microporous and Mesoporous Materials*.

[36] Jin Z., Zhao H. Y., Zhao X. J., Fang Q. R., Long J. R., Zhu G. S. (2010). A novel microporous MOF with the capability of selective adsorption of xylenes. *Chemical Communications*.

[37] Saccoccia B., Bohnsack A. M., Waggoner N. W. (2015). Separation of p-divinylbenzene by selective room-temperature adsorption inside Mg-CUK-1 prepared by aqueous microwave synthesis. *Angewandte Chemie, International Edition*.

[38] Peralta D., Chaplais G., Paillaud J. L., Simon-Masseron A., Barthelet K., Pirngruber G. D. (2013). The separation of xylene isomers by ZIF-8: a demonstration of the extraordinary flexibility of the ZIF-8 framework. *Microporous and Mesoporous Materials*.

[39] Vermoortele F., Maes M., Moghadam P. Z. (2011). *p*-Xylene-selective metal-organic frameworks: a case of topology-directed selectivity. *Journal of the American Chemical Society*.

[40] Moreira M. A., Santos J. C., Ferreira A. F. P. (2012). Toward understanding the influence of ethylbenzene in *p*-xylene selectivity of the porous titanium amino terephthalate MIL-125(Ti): adsorption equilibrium and separation of xylene isomers. *Langmuir*.

[41] Dang L. L., Zhang X. J., Zhang L., Li J. Q., Luo F., Feng X. F. (2016). Photo-responsive azo MOF exhibiting high selectivity for CO_2_ and xylene isomers. *Journal of Coordination Chemistry*.

[42] Rasouli M., Yaghobi N., Allahgholipour F., Atashi H. (2014). Para-xylene adsorption separation process using nano-zeolite Ba-X. *Chemical Engineering Research and Design*.

[43] Waitz T., Tsuchiya K., Antretter T., Fischer F. D. (2009). Phase transformations of nanocrystalline martensitic materials. *MRS Bulletin*.

[44] Chen C., Herhold A. B., Johnson C. S., Alivisatos A. P. (1997). Size dependence of structural metastability in semiconductor nanocrystals. *Science*.

[45] Wright J. S., Vitórica-Yrezábal I. J., Thompson S. P., Brammer L. (2016). Arene selectivity by a flexible coordination polymer host. *Chemistry - A European Journal*.

[46] Adams H., Batten S. R., Davies G. M. (2005). New bis-, tris- and tetrakis(pyrazolyl)borate ligands with 3-pyridyl and 4-pyridyl substituents: synthesis and coordination chemistry. *Dalton Transactions*.

