# Supplementary material for: Flexibility of Metal-Organic Framework Tunable by Crystal Size at the Micrometer to Submillimeter Scale for Efficient Xylene Isomer Separation
Source: Research (Wash D C). 2019 Oct 17;2019:9463719. doi: 10.34133/2019/9463719 (PMC6946284; doi:10.34133/2019/9463719)
Supplement: Supplementary Materials — Table S1: crystal-size-dependent framework flexibility reported for MOFs. Table S2: crystallographic data and structure refinements. Table S3: comparison of pX adsorption selectivities reported for MOFs. Figure S1: SEM images of A. Figure S2: crystal structure of A. Figure S3: detailed framework pore structures of A and B. Figure S4: TG curves of A. Figure S5: PXRD patterns of [Cu2(pypz)2]·0.5pX after DCM extraction. Figure S6: magnified Figure 2(d). Figure S7: magnified Figure 2(e). Figure S8: magnified Figure 2(f). Figure S9: the full illustration of the proposed transformation mechanism from B to C. Figure S10: optimized structures and energies of the structural fragments involved in the transformation from B to C. Figure S11: TG curves of 3B and 3C after adsorption of xylene isomers. Figure S12: PXRD patterns of 3B and 3C after having been loaded with xylene isomers. Figure S13: adsorption kinetic profiles of 3B and 3C for an equimolar mixture of xylene isomers. Figure S14: gas chromatography curve of the n-heptane solution containing an equimolar mixture of xylene isomers. Figure S15: xylene selectivities of 3B in three consecutive ad-/desorption cycles. Figure S16: xylene selectivities of 3C in three consecutive ad-/desorption cycles. Figure S17: TG curves for 1B after having been immersed in an equimolar mixture of xylene isomers for different times. Figure S18: xylene selectivities of 1B in three consecutive ad-/desorption cycles. [file 9463719.f1.pdf]

Supplementary Materials

Flexibility of Metal-Organic Framework Tunable by Crystal Size at the Micrometer to Submillimeter Scale for Efficient Xylene Isomer Separation

Xiao Yang, Hao-Long Zhou, Chun-Ting He, Zong-Wen Mo, Jia-Wen Ye, Xiao-Ming Chen, and Jie-Peng Zhang*

MOE Key Laboratory of Bioinorganic and Synthetic Chemistry, School of Chemistry, Sun Yat-Sen University, Guangzhou 510275, China

*zhangjp7@mail.sysu.edu.cn

Table of Contents

Table S1. Crystal-size dependent framework flexibility reported for MOFs

Table S2. Crystallographic data and structure refinements.

Table S3. Comparison of pX adsorption selectivities reported for MOFs.

Figure S1. SEM images of **A**.

Figure S2. Crystal structure of **A**.

Figure S3. Detailed framework pore structures of **A** and **B**.

Figure S4. TG curves of **A**.

Figure S5. PXRD patterns of $[Cu_2(pypz)_2]\cdot 0.5pX$ after DCM extraction.

Figure S6. Magnified Figure 2b.

Figure S7. Magnified Figure 2e.

Figure S8. Magnified Figure 2f.

Figure S9. The full illustration of the proposed transformation mechanism from **B** to **C**.

Figure S10. Optimized structures and energies of the structural fragments involved in the transformation from **B** to **C**.

Figure S11. TG curves of **3B** and **3C** after adsorption of xylene isomers.

Figure S12. PXRD patterns of **3B** and **3C** after loaded with xylene isomers.

Figure S13. Adsorption kinetic profiles of **3B** and **3C** for equimolar mixture of xylene isomers.

Figure S14. Gas chromatography curve of the *n*-heptane solution containing equimolar mixture of xylene isomers.

Figure S15. Xylene selectivities of **3B** in three consecutive ad-/desorption cycles.

Figure S16. Xylene selectivities of **3C** in three consecutive ad-/desorption cycles.

Figure S17. TG curves for **1B** after immersed in equimolar mixture of xylene isomers for different times.

Figure S18. Xylene selectivities of **1B** in three consecutive ad-/desorption cycles.

References

Table S1. Crystal-size dependent framework flexibility reported for MOFs.

MOF	crystal size range	Downsizing effect	reference
$[Cu_2(pypz)_2]$	500 μm ~ 1 μm	The phase transition temperature from the metastable guest-free phase (**B**) to the stable guest-free phase (**C**) increases.	This work
MAF-4 (ZIF-8)	15.8 μm ~ 10 nm	The second step of gas adsorption isotherm starts at higher pressure.	[1, 2]
DUT-49	4.08 μm ~ 100 nm	The pressure amplifications in the *n*-butane and N_2 adsorption isotherms gradually vanish.	[3]
$[Cu_2(bdc)_2(bpe)]$	700 nm ~ 50 nm	The guest removal-induced open to close framework shrinkage gradually vanishes.	[4]
DUT-8(Ni)	500 nm	The guest removal-induced open to close framework shrinkage vanishes.	[5]
$[Cu_2(bdc)_2(bpy)]$	160 nm ~ 50 nm	The guest removal-induced open to close framework shrinkage gradually vanishes.	[4]
$[Pt(CN)_4Fe(py)_2]$	16 nm	From no adsorption to gate-opening behavior.	[6]

Table S2. Crystallographic data and structure refinements.

compound	**A**	**B**	**C**
formula	$C_{20}H_{17}Cu_2N_6$	$C_{16}H_{12}Cu_2N_6$	$C_{16}H_{12}Cu_2N_6$
FW	468.47	415.40	415.40
T (K)	150(2)	150(2)	150(2)
space group	$P2_1/c$	$P2_1/c$	$P2_1/c$
a (Å)	5.71894(10)	5.3901(3)	5.2359(1)
b (Å)	20.3635(3)	20.5512(6)	15.3176(5)
c (Å)	15.3915(2)	15.2289(6)	19.5258(6)
β/(°)	94.0980(14)	98.504(4)	93.307(3)
V/(Å^3)	1787.88(5)	1668.4(1)	1563.37(8)
Z	4	4	4
D_c/g cm^{-3}	1.740	1.654	1.765
μ (mm^{-1})	3.085	3.218	3.435
reflns coll.	15562	12581	10113
unique reflns.	3686	3059	2755
R_{int}	0.0180	0.0478	0.0867
R_1 (> 2σ(I)) ^{a}	0.0279	0.0643	0.0669
wR_2 (> 2σ(I)) ^{b}	0.0779	0.2005	0.1700
R_1 (all data) ^{a}	0.0292	0.0724	0.0859
wR_2 (all data) ^{b}	0.0843	0.2103	0.1854
GOF	1.022	1.094	1.055

^{a} $R_1 = \sum||F_o|-|F_c||/\sum|F_o|$, ^{b} $wR_2 = [\sum w(F_o^2-F_c^2)^2/\sum w(F_o^2)^2]^{1/2}$

Table S3. Comparison of pX adsorption selectivities reported for MOFs.

MOF	α_{pXoX}	α_{pXmX}	$\alpha_{pX/(oX + mX)}$	component	reference
**3C**	53.9	48.9	51.3	ternary	This work
**3B**	17	15	16	ternary	This work
**1B**	16	14	15	ternary	This work
$Ag_4(O_2CCF_3)_4(phen)_3$	9.13[a]	14.2[a]	NA	binary	[7]
Cu(CDC)	10	7	NA	binary	[8]
[Nd(HTCPB)]	NR	6.33	NA	binary	[9]
[Ce(HTCPB)]	5.65	4.55	NA	binary	[9]
MIL-125(Ti)_NH_2	2.2	4.4	NA	binary	[10]
MIL-125(Ti)	NR	3.5	NA	binary	[10]
[Zn(μ_4-L)]	3.2	2.7	2.9	ternary	[11]
MAF-4 (ZIF-8)	3.1	NR	NA	binary	[12]
CAU-1(Al)-NH_2	NR	2.8	NA	binary	[10]
MIL-140B	1.8	1.6	1.7	quaternary	[13]
MOF-48	1.7	1.7	1.7	quaternary	[13]

[a] This is not adsorption selectivity. The guests were included during the synthesis of the framework.

NR: Not reported

NA: Not available
phen = phenazine

H_2CDC = *trans*-1, 4-cyclohexanedicarboxylic acid

H_2TCPB = 4’,5’-bis(4-carboxyphenyl)-[1,1’,2’,1’’-terphenyl]-4,4’’-dicarboxylic acid

H_2L = biphenyl-3,5-dicarboxylic acid

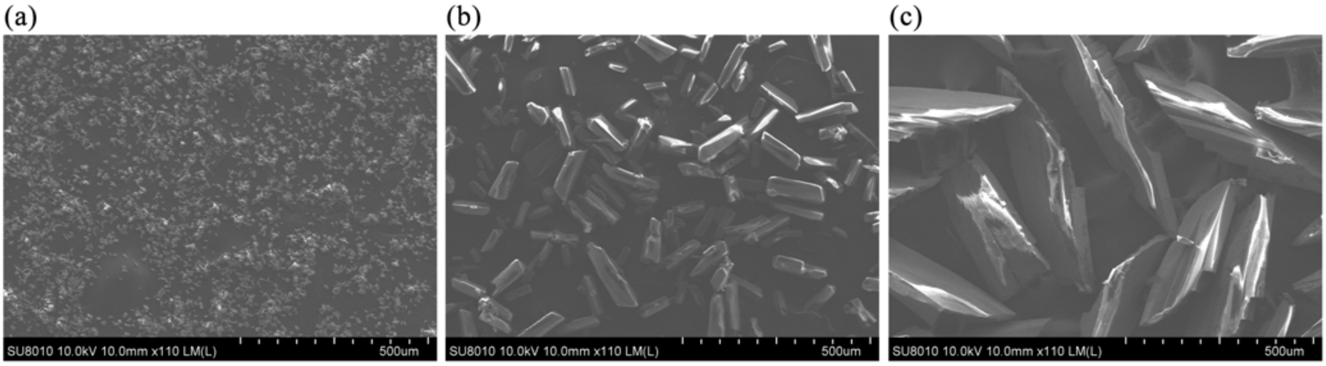

Figure S1. SEM images of **A**. (a) **1**, (b) **2**, and (c) **3**.

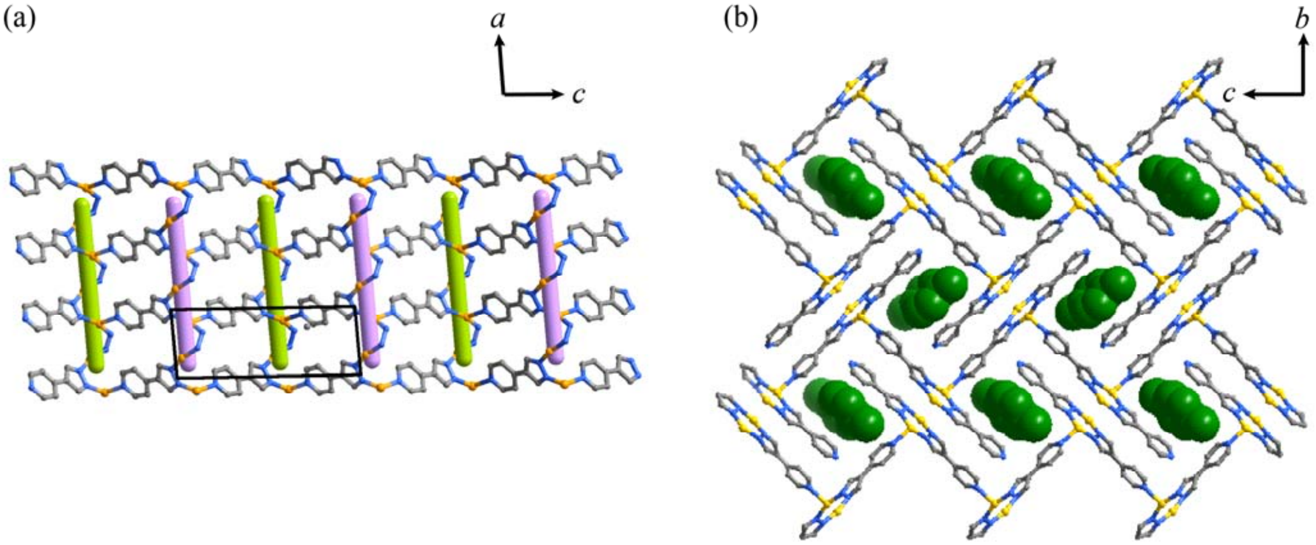

Figure S2. Crystal structure of **A**: (a) a zigzag coordination layer (adjacent helical chains are highlighted in olive and violet); (b) the packing structure along the channel direction (*p*-xylene molecules are shown in space-filling mode and highlighted in green).

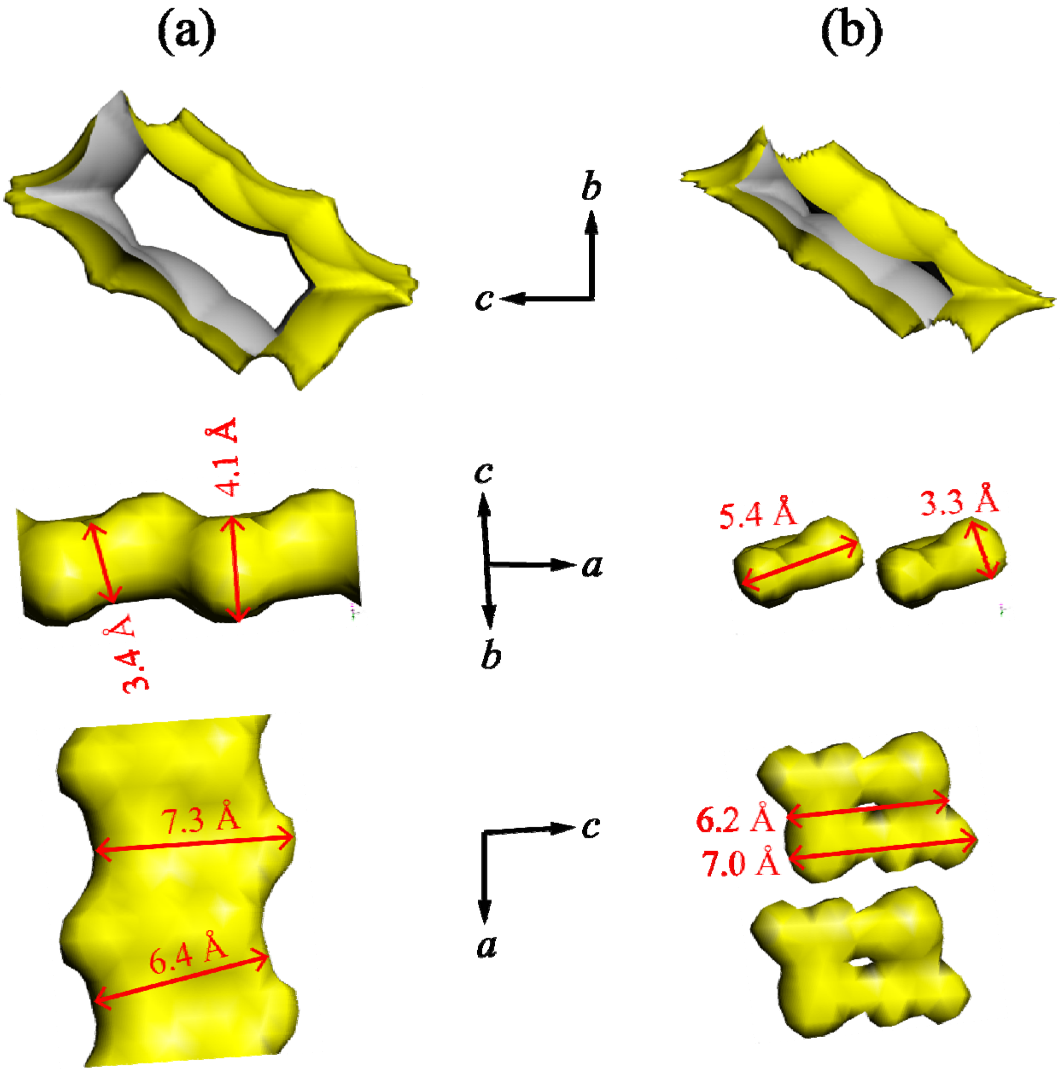

Figure S3. Detailed pore structures of (a) **A** and (b) **B** viewing along three typical directions (the pores shown in the top pannels have are shown in the same direction as in Figure 3).

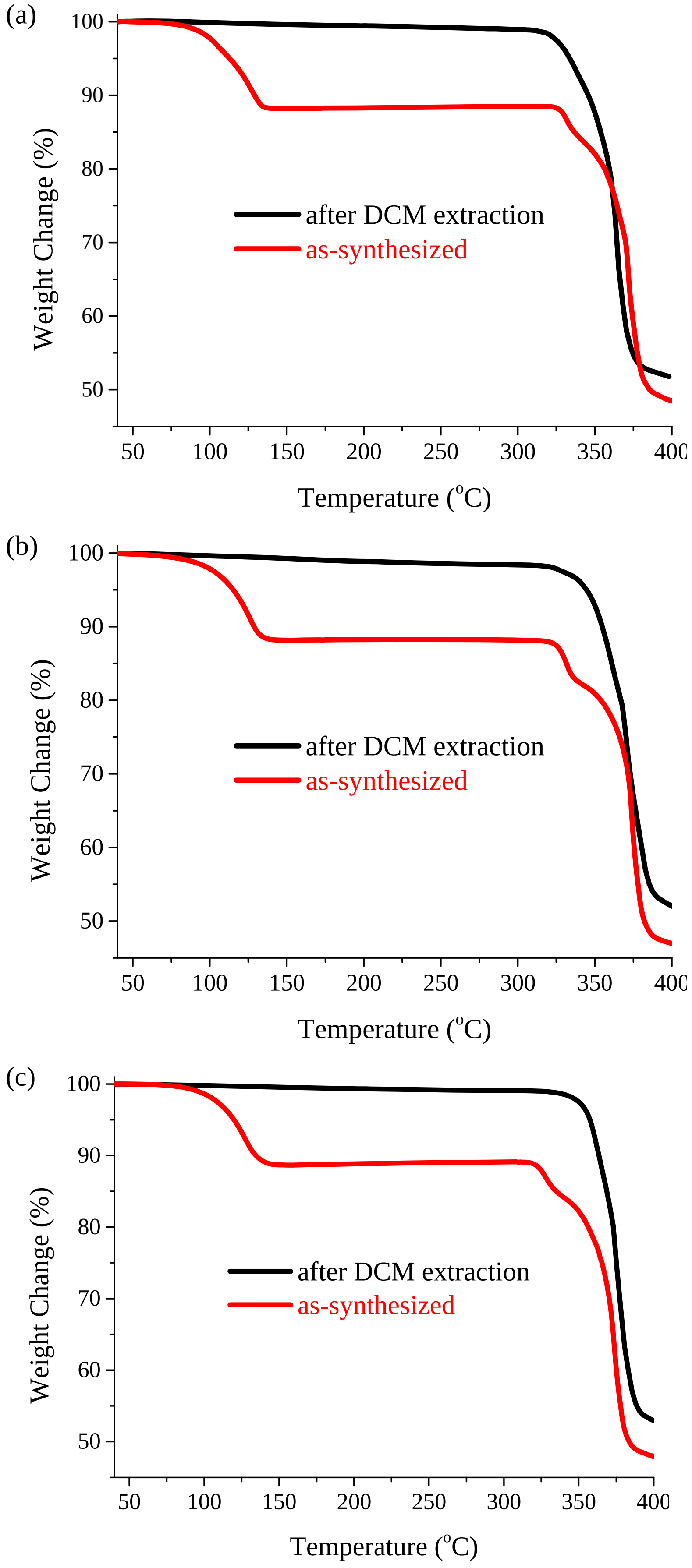

Figure S4. TG curves of (a) **1**, (b) **2**, and (c) **3**.

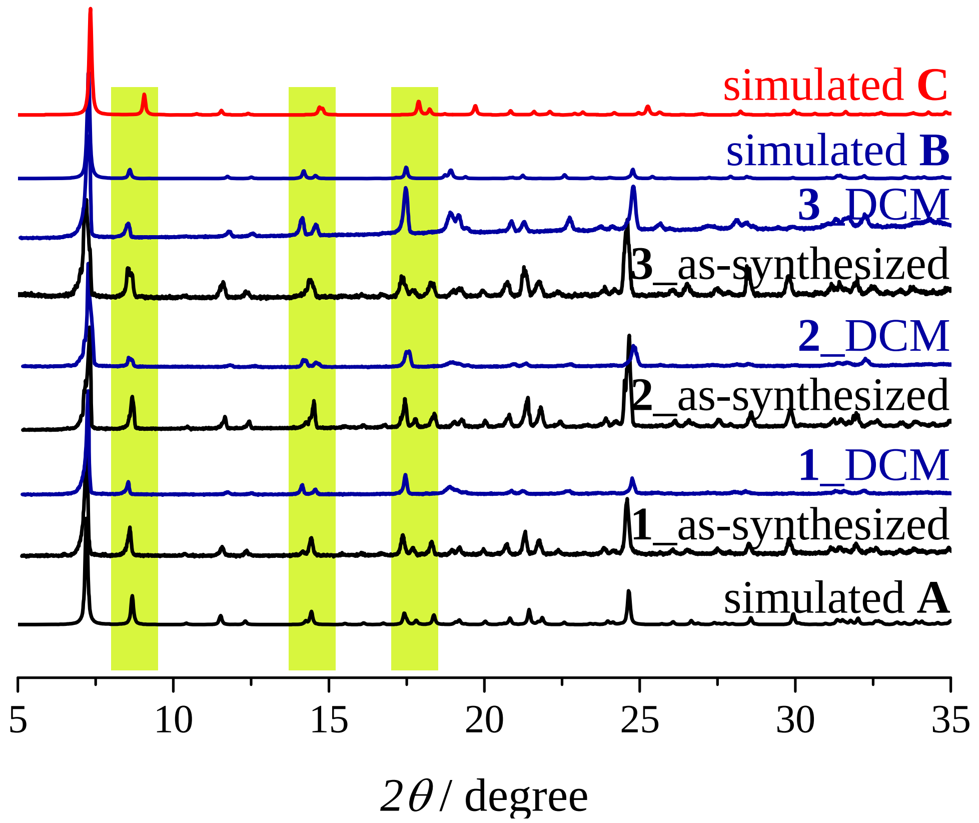

Figure S5. PXRD patterns of $[Cu_2(pypz)_2]\cdot 0.5pX$ (**1**, **2**, **3**) after DCM extraction.

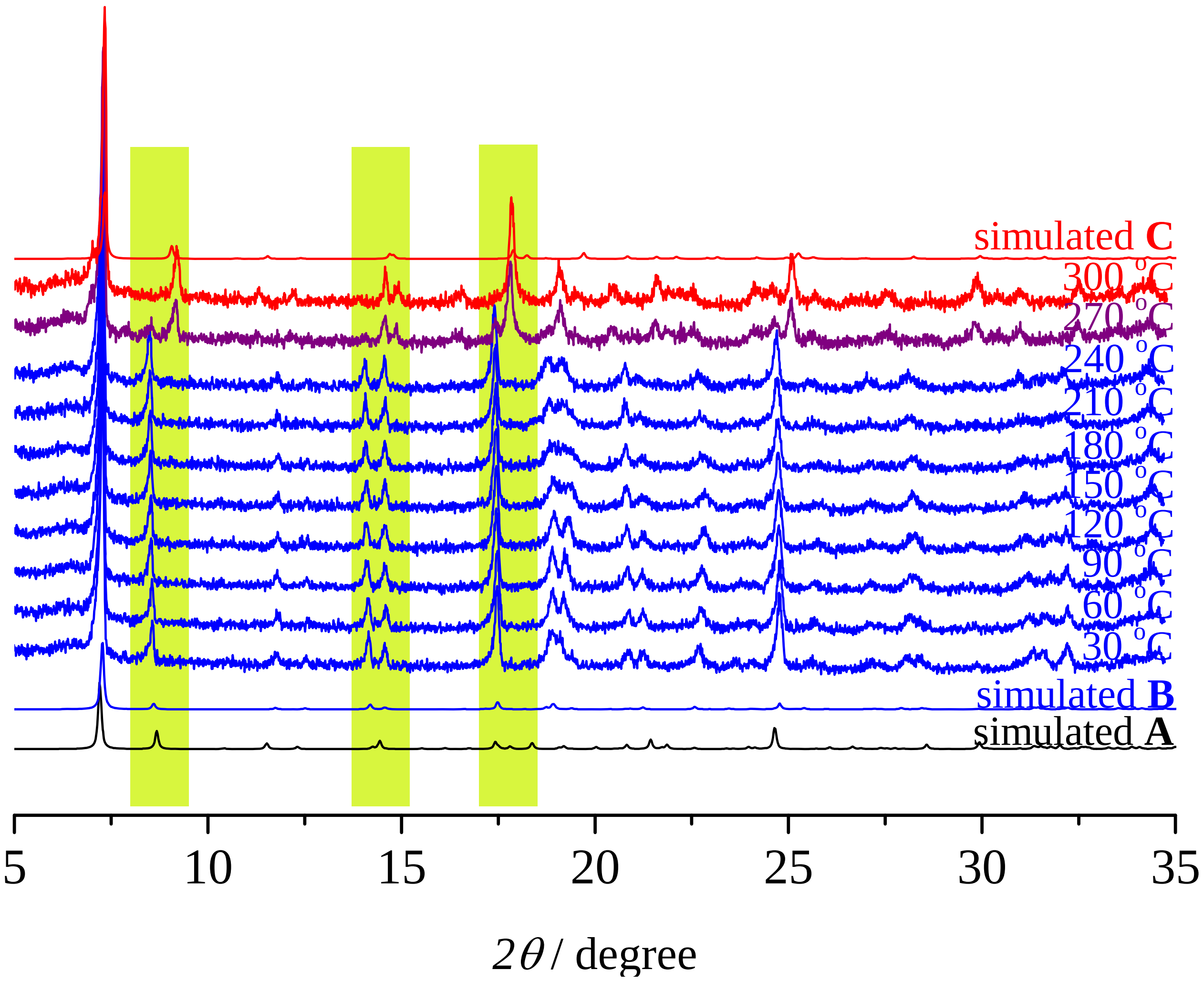

Figure S6. Magnified Figure 2d.

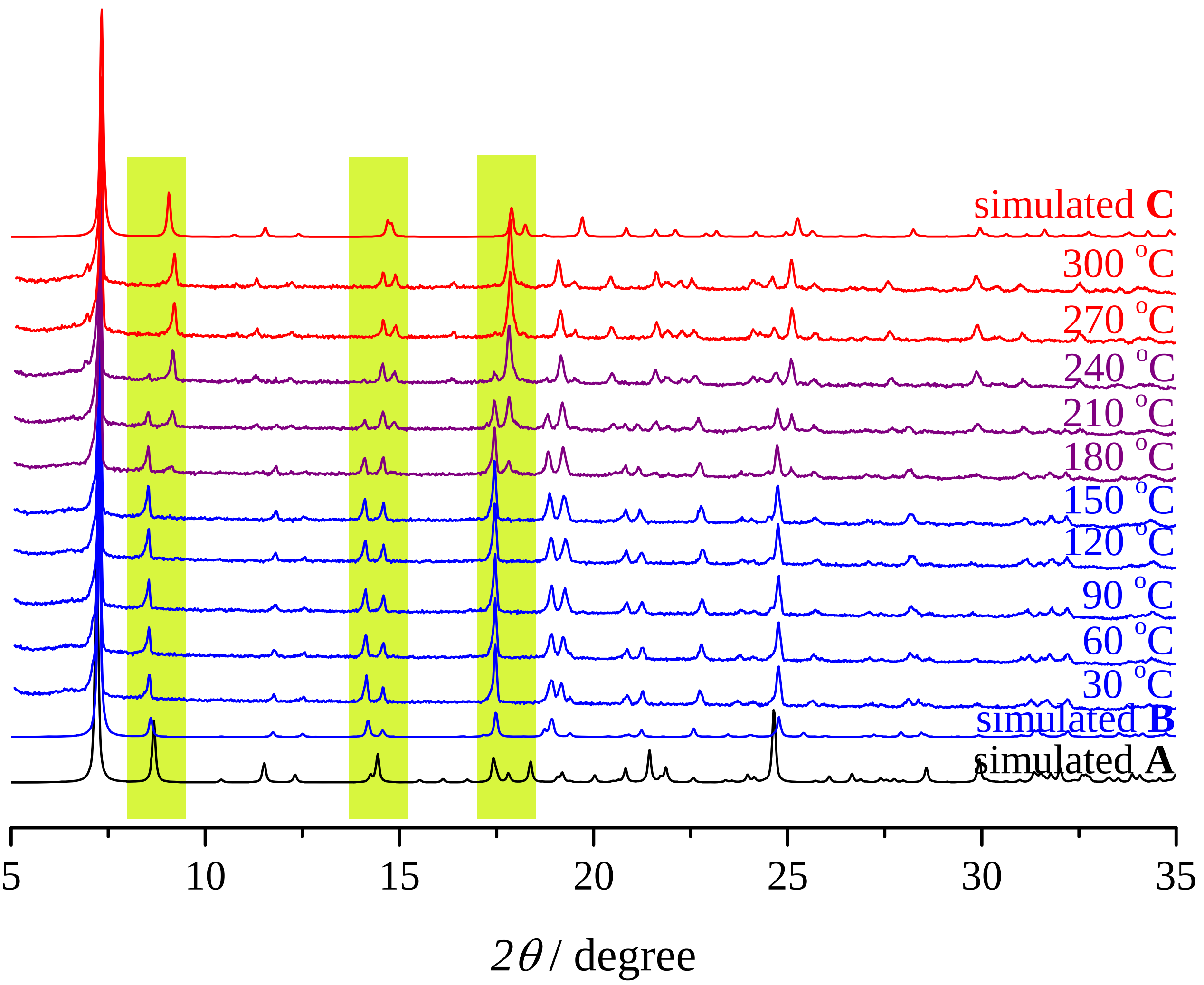

Figure S7. Magnified Figure 2e.

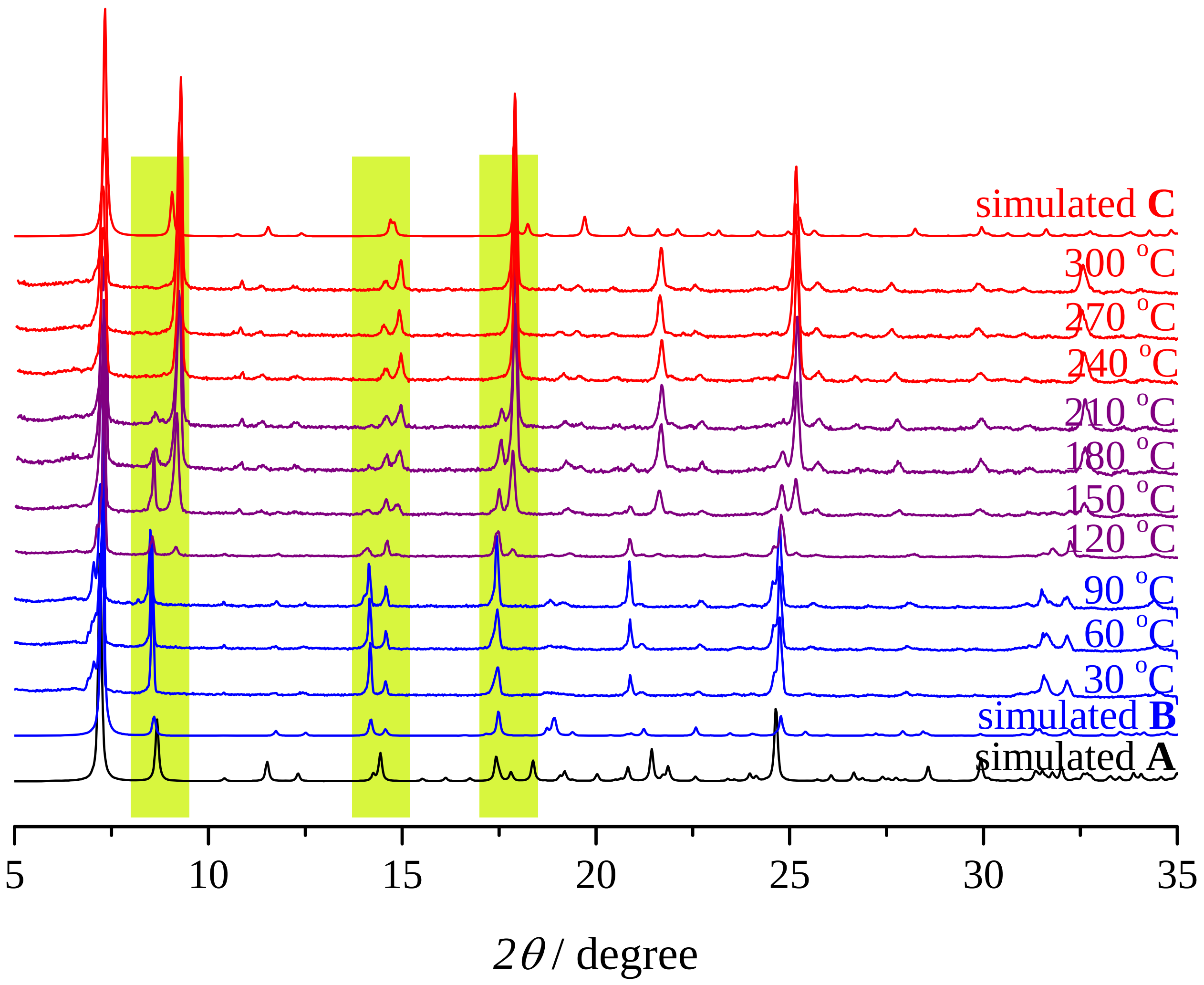

Figure S8. Magnified Figure 2f.

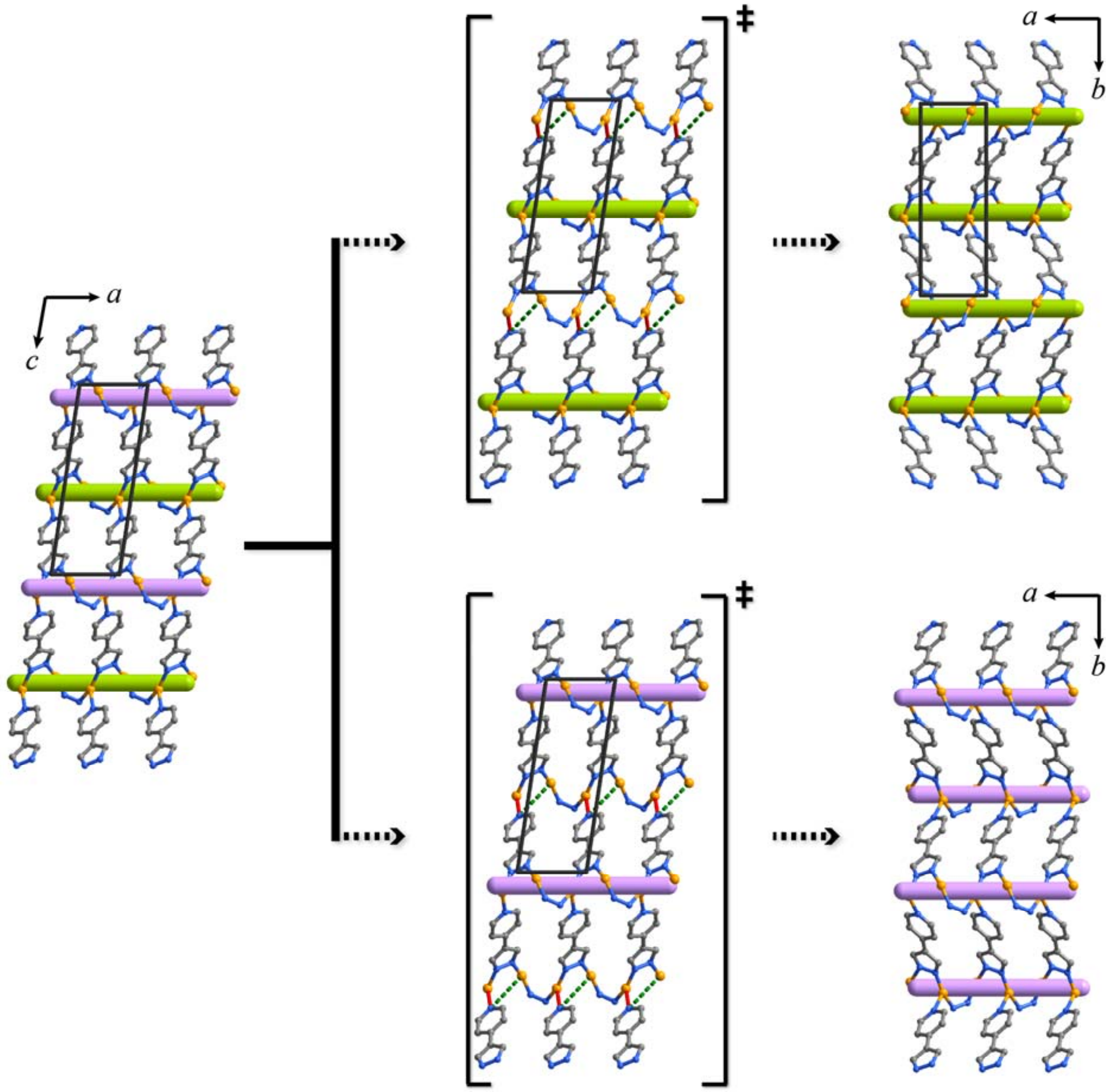

Figure S9. The full illustration of the proposed transformation mechanism from **B** to **C**.

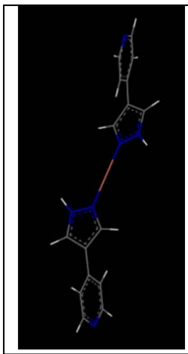	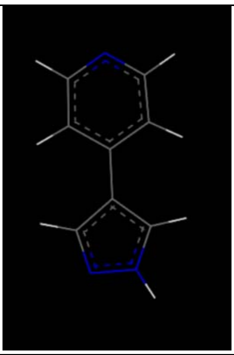	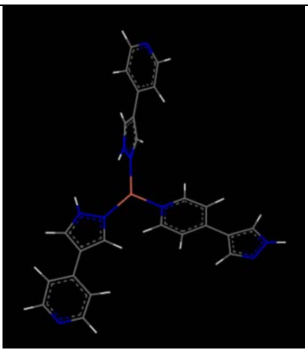
E_1 = -1168.1218953 Ha	E_2 = -472.8931741 Ha	E_3 = -1641.0536590 Ha
$\Delta E = E_1+E_2-E_3$ = 101.3 kJ/mol		

Figure S10. Optimized and energies of the structural fragments involved in the transformation from **B** to **C**.

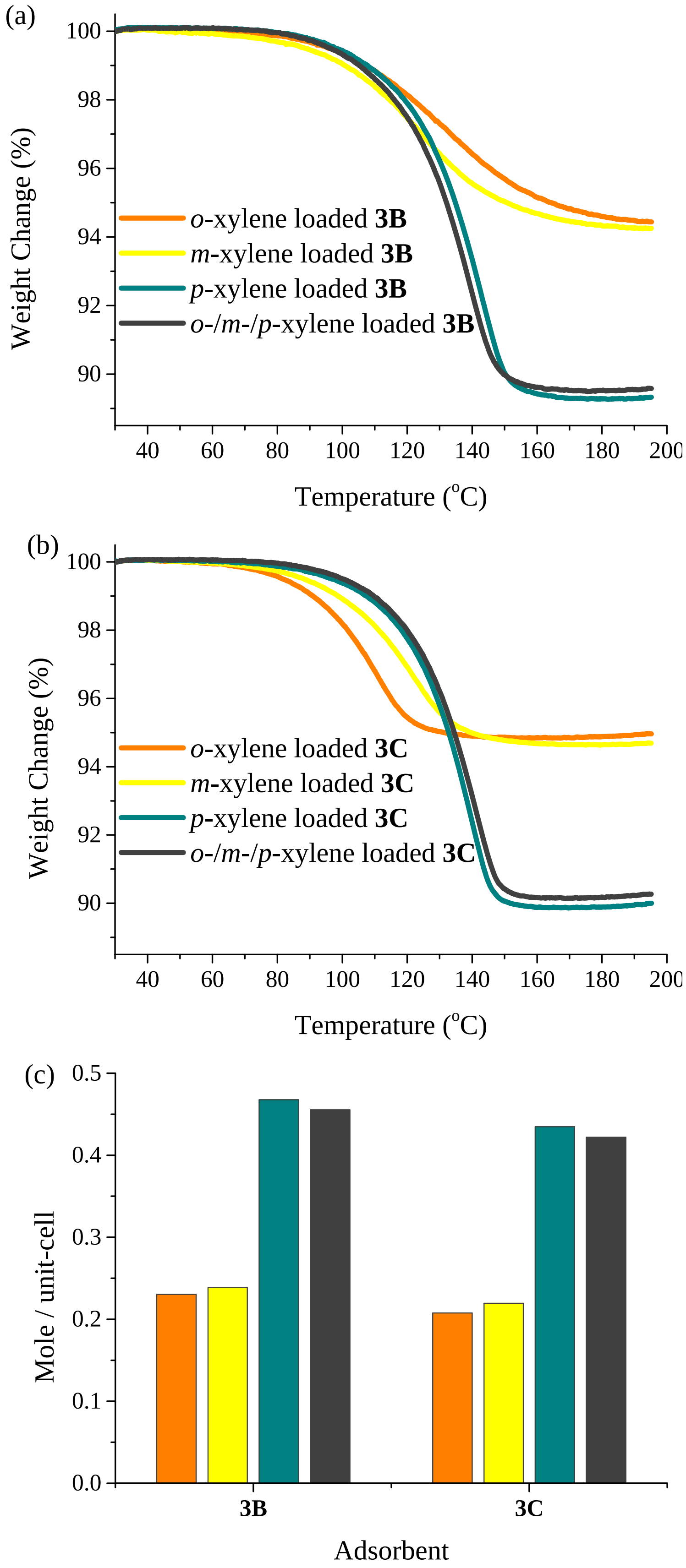

Figure S11. TG curves of (a) **3B** and (b) **3C** after adsorption of xylene isomers. (c) Summary of xylene uptakes. Colour scheme: *o*-xylene, orange; *m*-xylene, yellow; *p*-xylene, dark cyan; ternary xylene mixture, dark grey.

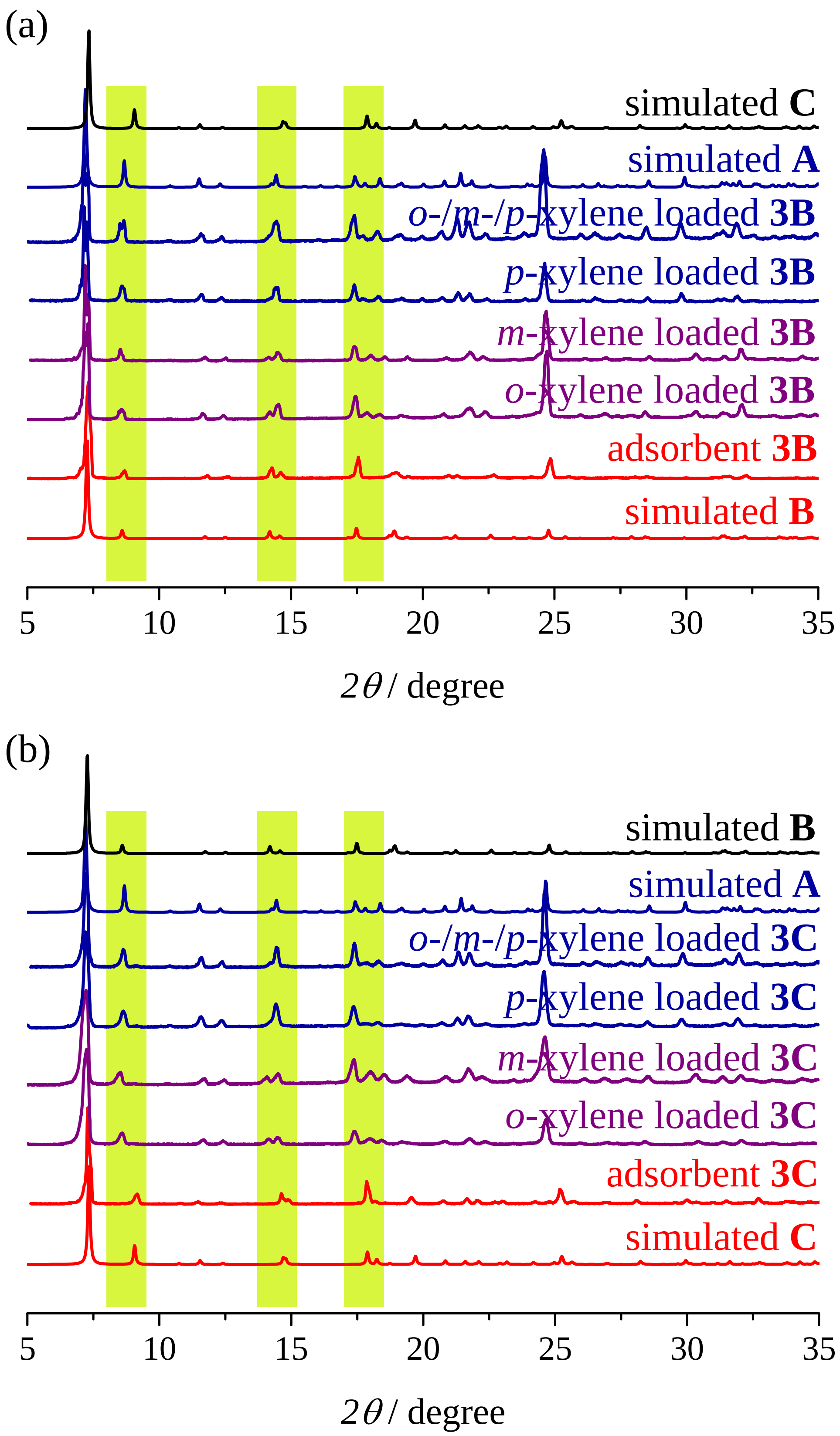

Figure S12. PXRD patterns of (a) **3B** and (b) **3C** after loaded with xylene isomers.

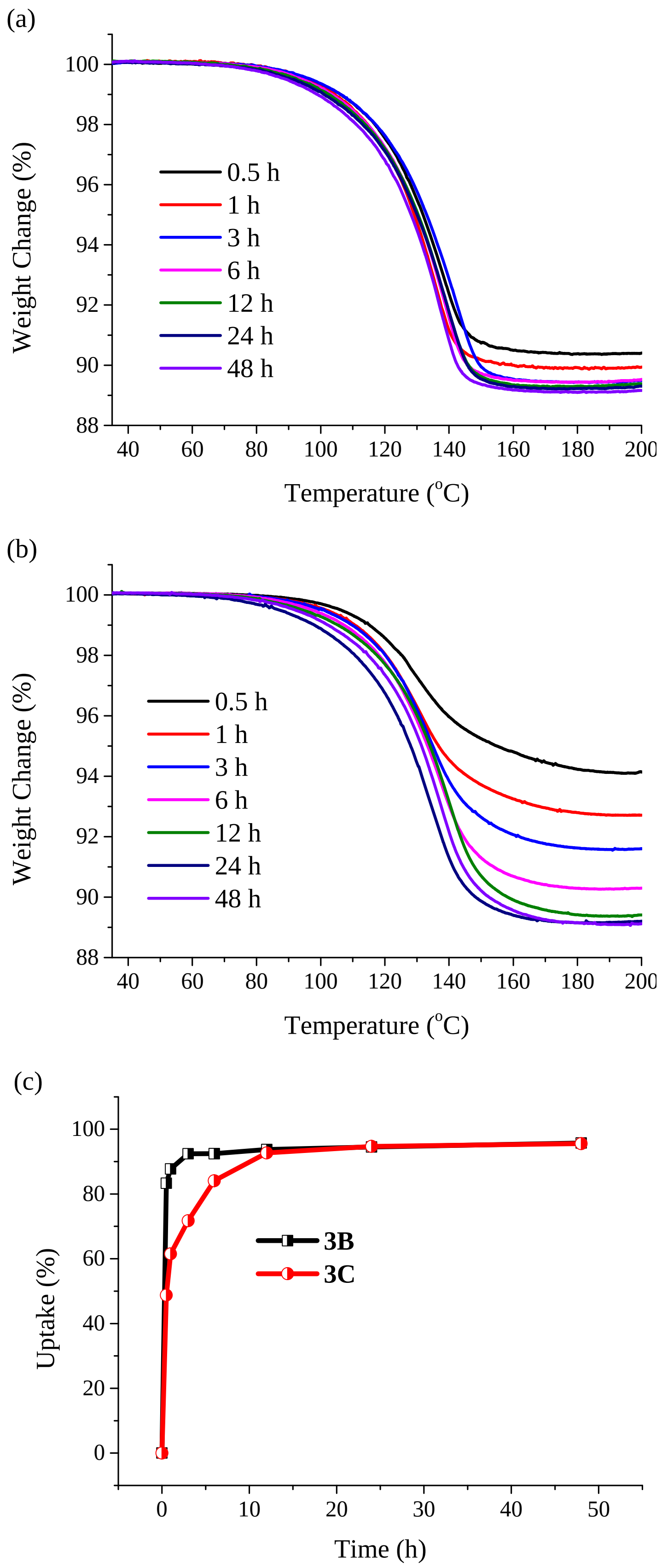

Figure S13. TG curves for (a) **3B** and (b) **3C** after immersion into equimolar mixture of xylene isomers for different times. (c) The corresponding adsorption kinetic profiles.

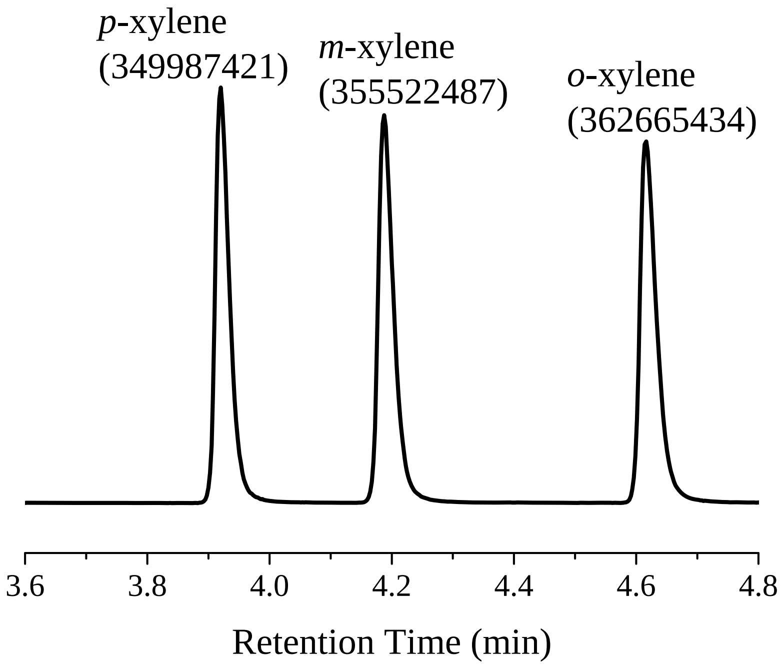

Figure S14. Gas chromatography curve of the *n*-heptane solution containing equimolar ternary mixture of xylene isomers. The integrated peak areas have been added in the parentheses above their corresponding peaks.

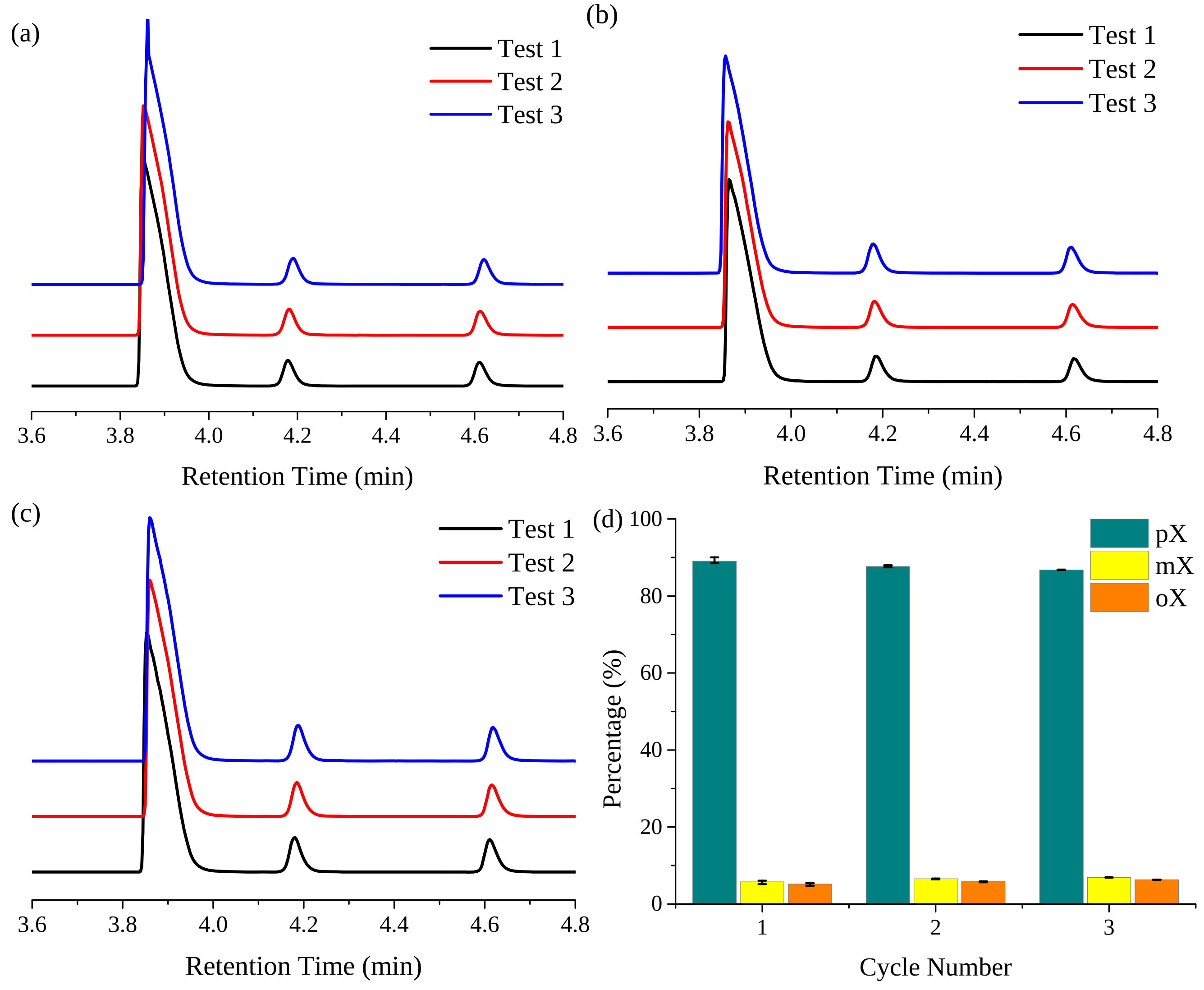

Figure S15. Xylene selectivities of **3B** in three consecutive ad-/desorption cycles. Gas chromatography curves of the digestion solutions of **3B** samples after immersed in equimolar mixture of xylene isomers in the (a) first, (b) second, and (c) third ad-/desorption cycle. Three parallel tests have been performed for each cycle. (d) Relative xylene isomer uptake in **3B** for three consecutive ad-/desorption cycles.

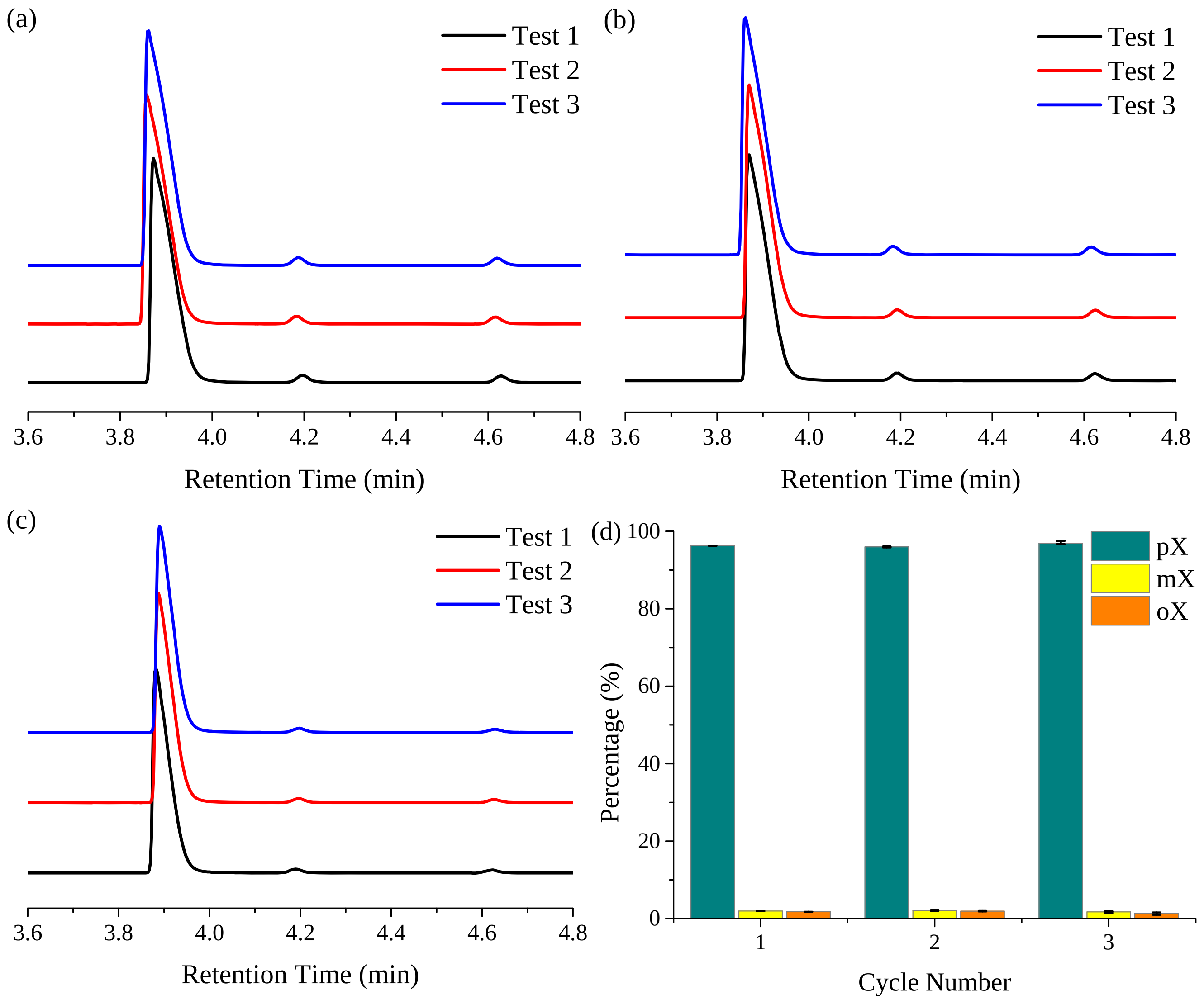

Figure S16. Xylene selectivities of **3C** in three consecutive ad-/desorption cycles. Gas chromatography curves of the solutions of **3C** samples after immersed in equimolar mixture of xylene isomers in the (a) first, (b) second, and (c) third ad-/desorption cycle. Three parallel tests have been performed for each cycle. (d) Relative xylene isomer uptake in **3C** for three consecutive ad-/desorption cycles.

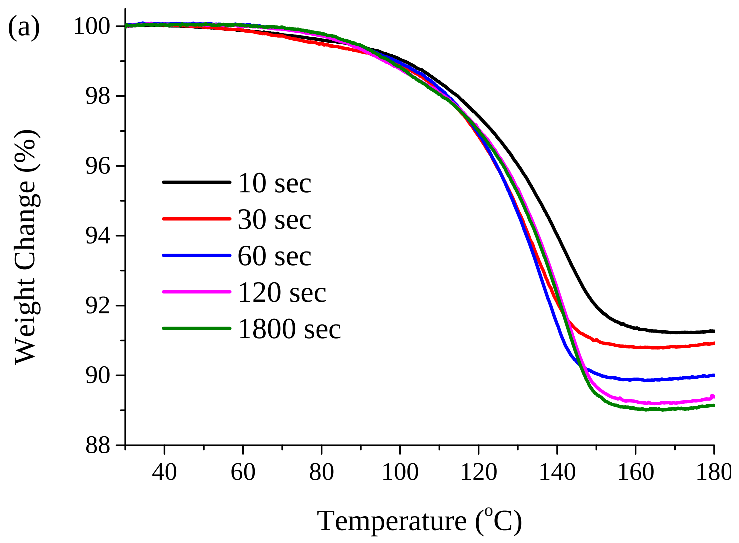

Figure S17. TG curves for **1B** after immersed in equimolar mixture of xylene isomers for different times.

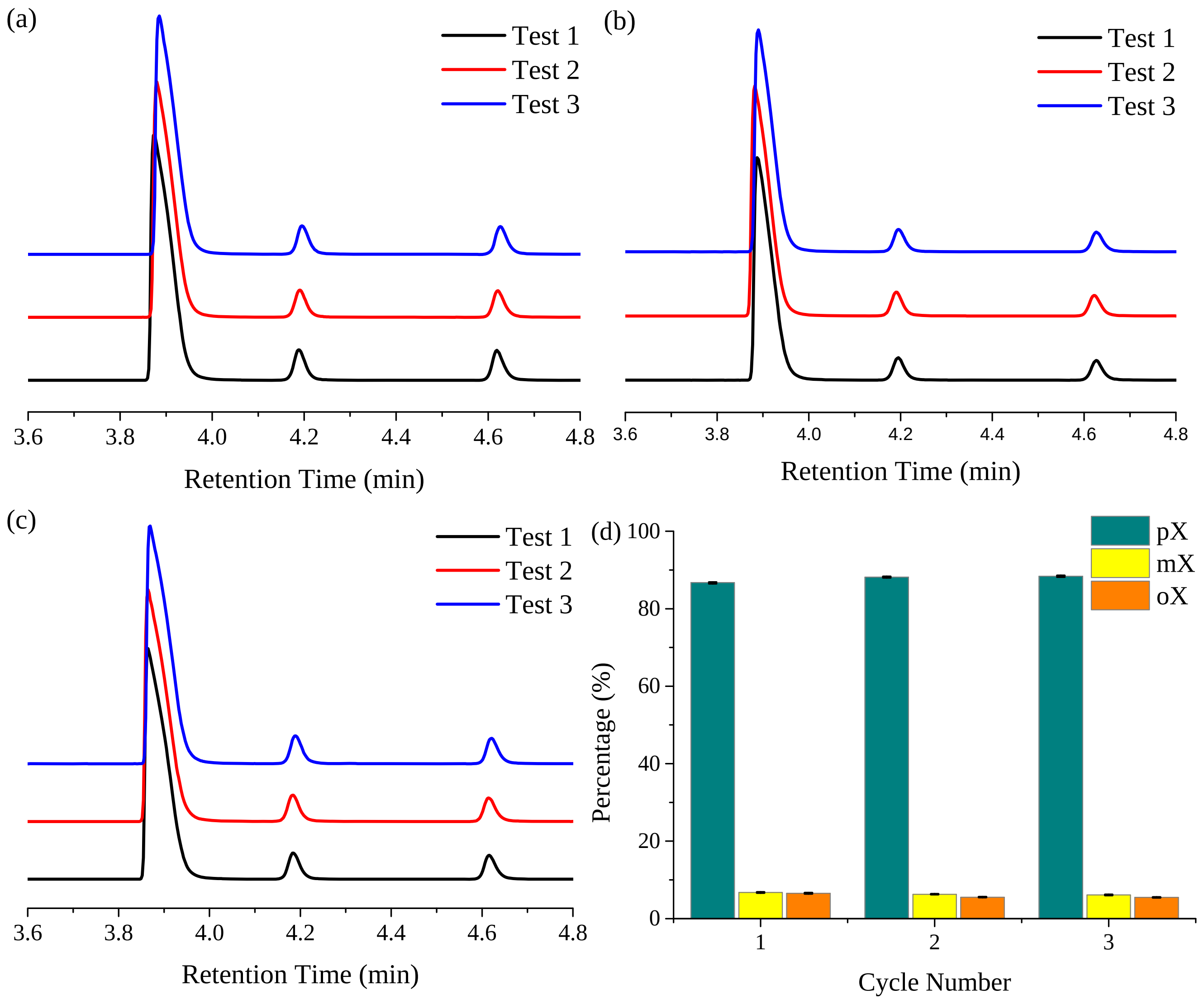

Figure S18. Xylene selectivities of **1B** in three consecutive ad-/desorption cycles. Gas chromatography curves of the digestion solutions of **1B** samples after immersed in equimolar mixture of xylene isomers in the (a) first, (b) second, and (c) third ad-/desorption cycle. Three parallel tests have been performed for each cycle. (d) Relative xylene isomer uptake in **1B** for three consecutive ad-/desorption cycles.

References

[1] S. Tanaka, K. Fujita, Y. Miyake, M. Miyamoto, Y. Hasegawa, T. Makino, S. Van der Perre, J. Cousin Saint Remi, T. Van Assche, G. V. Baron, and J. F. M. Denayer, "Adsorption and diffusion phenomena in crystal size engineered ZIF-8 MOF," *J. Phys. Chem. C*, vol. 119, no. 51, pp. 28430-28439, 2015.

[2] C. Zhang, J. A. Gee, D. S. Sholl, and R. P. Lively, "Crystal-size-dependent structural transitions in nanoporous crystals: adsorption-induced transitions in ZIF-8," *J. Phys. Chem. C*, vol. 118, no. 35, pp. 20727-20733, 2014.

[3] S. Krause, V. Bon, I. Senkovska, D. M. Tobbens, D. Wallacher, R. S. Pillai, G. Maurin, and S. Kaskel, "The effect of crystallite size on pressure amplification in switchable porous solids," *Nat. Commun.*, vol. 9, no. 1, pp. 1573, 2018.

[4] Y. Sakata, S. Furukawa, M. Kondo, K. Hirai, N. Horike, Y. Takashima, H. Uehara, N. Louvain, M. Meilikhov, T. Tsuruoka, S. Isoda, W. Kosaka, O. Sakata, and S. Kitagawa, "Shape-memory nanopores induced in coordination frameworks by crystal downsizing," *Science*, vol. 339, no. 6116, pp. 193-196, 2013.

[5] N. Kavoosi, V. Bon, I. Senkovska, S. Krause, C. Atzori, F. Bonino, J. Pallmann, S. Paasch, E. Brunner, and S. Kaskel, "Tailoring adsorption induced phase transitions in the pillared-layer type metal-organic framework DUT-8(Ni)," *Dalton Trans.*, vol. 46, no. 14, pp. 4685-4695, 2017.

[6] Sakaida, S., K. Otsubo, O. Sakata, C. Song, A. Fujiwara, M. Takata, and H. Kitagawa, "Crystalline coordination framework endowed with dynamic gate-opening behaviour by being downsized to a thin film," *Nat. Chem.*, vol. 8, no. 4, pp. 377-83, 2016.

[7] J. S. Wright, I. J. Vitórica-Yrezábal, S. P. Thompson, and L. Brammer, "Arene selectivity by a flexible coordination polymer host," *Chem. Eur. J.*, vol. 22, no. 37, pp. 13120-13126, 2016.

[8] J. Lannoeye, B. Van de Voorde, B. Bozbiyik, H. Reinsch, J. F. M. Denayer, and D. De Vos, "An aliphatic copper metal-organic framework as versatile shape selective adsorbent in liquid phase separations," *Micropor. Mesopor. Mater.*, vol. 226, pp. 292-298, 2016.

[9] J. E. Warren, C. G. Perkins, K. E. Jelfs, P. Boldrin, P. A. Chater, G. J. Miller, T. D. Manning, M. E. Briggs, K. C. Stylianou, J. B. Claridge, and M. J. Rosseinsky, "Shape selectivity by guest-driven restructuring of a porous material," *Angew. Chem. Int. Ed.*, vol. 53, no. 18, pp. 4592-4596, 2014.

[10] F. Vermoortele, M. Maes, P. Z. Moghadam, M. J. Lennox, F. Ragon, M. Boulhout, S. Biswas, K. G. Laurier, I. Beurroies, R. Denoyel, M. Roeffaers, N. Stock, T. Duren, C. Serre, and D. E. De Vos, "p-Xylene-selective metal-organic frameworks: a case of topology-directed selectivity," *J. Am. Chem. Soc.*, vol. 133, no. 46, pp. 18526-18529, 2011.

[11] W. Huang, J. Jiang, D. Wu, J. Xu, B. Xue, and A. M. Kirillov, "A highly stable nanotubular MOF rotator for selective adsorption of benzene and separation of xylene isomers," *Inorg. Chem.*, vol. 54, no. 22, pp. 10524-10526, 2015.

[12] Peralta, D., G. Chaplais, A. Simon-Masseron, K. Barthelet, C. Chizallet, A. A. Quoineaud, and G. D. Pirngruber, "Comparison of the behavior of metal-organic frameworks and zeolites for hydrocarbon separations," *J. Am. Chem. Soc.*, vol. 134, no. 19, pp. 8115-26, 2012.

[13] J. A. Gee, K. Zhang, S. Bhattacharyya, J. Bentley, M. Rungta, J. S. Abichandani, D. S. Sholl, and S. Nair., "Computational identification and experimental evaluation of metal-organic frameworks for xylene enrichment," *J. Phys. Chem. C*, vol. 120, pp. 12075-12082, 2016.
